# Quantum scale biomimicry of low dimensional growth: An unusual complex amorphous precursor route to TiO_2_ band confinement by shape adaptive biopolymer-like flexibility for energy applications

**DOI:** 10.1038/s41598-019-55103-z

**Published:** 2019-12-10

**Authors:** Dahyun Choi, Sanjiv Sonkaria, Stephen J. Fox, Shivraj Poudel, Sung-yong Kim, Suhee Kang, Seheon Kim, Chandra Verma, Sung Hoon Ahn, Caroline Sunyong Lee, Varsha Khare

**Affiliations:** 1 Department of Materials Engineering, Hanyang University, Ansan, 15588 Republic of Korea; 2 Institute of Advanced Machinery and Design, Seoul National University, Daehak-dong, Gwanak-gu, Seoul 151-742 Republic of Korea; 3 Bioinformatics Institute (A*STAR), 30 Biopolis Street, #07-01 Matrix, Singapore, 138671 Singapore; 4 Department of Biological Sciences, National University of Singapore, 16 Science Drive, Singapore, 117558 Singapore; 5 School of Biological Sciences, Nanyang Technological University, 60 Nanyang Drive, Singapore, 637551 Singapore; 6 Department of Mechanical and Aerospace Engineering, Seoul National University, Daehak-dong, Gwanak-gu, Seoul 08826 Republic of Korea

**Keywords:** Energy science and technology, Materials science

## Abstract

Crystallization via an amorphous pathway is often preferred by biologically driven processes enabling living species to better regulate activation energies to crystal formation that are intrinsically linked to shape and size of dynamically evolving morphologies. Templated ordering of 3-dimensional space around amorphous embedded non-equilibrium phases at heterogeneous polymer─metal interfaces signify important routes for the genesis of low-dimensional materials under stress-induced polymer confinement. We report the surface induced catalytic loss of P=O ligands to bond activated aromatization of C−C C=C and Ti=N resulting in confinement of porphyrin-TiO_2_ within polymer nanocages via particle attachment. Restricted growth nucleation of TiO_2_ to the quantum scale (≤2 nm) is synthetically assisted by nitrogen, phosphine and hydrocarbon polymer chemistry via self-assembly. Here, the amorphous arrest phase of TiO_2_ is reminiscent of biogenic amorphous crystal growth patterns and polymer coordination has both a chemical and biomimetic significance arising from quantum scale confinement which is atomically challenging. The relative ease in adaptability of non-equilibrium phases renders host structures more shape compliant to congruent guests increasing the possibility of geometrical confinement. Here, we provide evidence for synthetic biomimicry akin to bio-polymerization mechanisms to steer disorder-to-order transitions via solvent plasticization-like behaviour. This challenges the rationale of quantum driven confinement processes by conventional processes. Further, we show the change in optoelectronic properties under quantum confinement is intrinsically related to size that affects their optical absorption band energy range in DSSC.

## Introduction

Quantum confinement is intimately connected with material surface properties and nucleation events. The fabrication of low-dimensional materials at the sub-nanometer scale continues to be a major driving force to improve semiconductor physics and device efficiency. TiO_2_ is intrinsically ‘locked’ within a wide band-gap photon absorption range substantially limiting its sensitivity to UV and IR solar emissions. This poses a technological barrier in harnessing the broader solar spectrum of emitted electromagnetic waves during incident–photon absorption. The quantum confinement effect has important consequences in decoupling ‘dimensionality’ from the bulk properties allowing band gap and band edge to become tunable entities^1^. Semiconductor dynamics of shape^2^ and size control in the quantum state are therefore critical parameters in increasing the availability of photo-generated charge carriers during photoexcitation.

The unique fundamental characteristics of low-dimensional materials bring ‘new’ synthetic challenges to their fabrication — TiO_2_ is a *philosophical* example of the difficulties in configuring a quantum state of matter which has been extremely ‘hard-to-achieve’ synthetically. A more exciting and insightful perspective in improving size-control‘ of metals, semiconductors and their complexes is being born from the principles biomineralisation. Bio-mediated semiconductor synthesis^3^ permits access to kinetic pathways to form quantum confined assemblies^4,5^ that may otherwise be hindered by conventional synthetic mechanisms. However, the pioneering work of Prof. Mann has been insightful in directing exploratory lines towards a better understanding of the connections that exist between chemistry and biology while building a body of awareness of how bio-driven hierarchical structures have relevancy to synthetic ones^6,7^. An area of biomimetic importance are self-assembled structures under confinement identified in organisms. A more comprehensive understanding of synthetically driven counterparts is slowly emerging as a compelling area of research^8,9^.

Some insight has been gained from microbially assembled biopolymers which use storage polyesters to drastically slow down crystallization of the nucleating phase which primarily stems from their amorphous nature *in vivo*^10^. Confinement effects alone have proved to be important regulators in delaying amorphous calcium carbonate (ACC) crystalline transitions^11^ while polyanionic matrix protein such as caspartin from the mollusk shell forms a polymerizable honey-comb-like envelope geometrically restricting calcite outgrowth^12^. Intervention by polystyrene sulphonate^13^, phosphinic^14^ and alkylphosphonic^15^ surface ligands reportedly result in both size and morphological control of respective amorphous and crystal states by growth inhibition. An interesting scenario that has much relevance to the chemical physics of protein folding^16^ is the ‘amorphous state’ — preassigned as the nucleation precursor to crystallization^17^. Such intermediates have overlapping significance with the pre-crystallization phase of the biomineralized amorphous state. The transient nature of folding assemblies and surface driven landscapes and non-equilibrium amorphous pathways provide ‘natures’ opportunity to tune both the size and shape of bio-structures through a series of trapped conformational and competing non-equilibrium polymorphic states^18^.

The biological observations documented above provide a number of critical clues towards implementing synthetic mimicry at the quantum scale that may be of general applicability to different material types. At the core of the problem lies ‘surface dynamics’ and the need to steer interfacial forces with programmable precision to select for key pathways that slow nucleation growth. This suggests that the origins of molecular self-assembly must lie at the interface of contrasting materials reshaping landscape energies at the surface of nucleating species. This interpretation is supported computationally by non-classical^19^ biogenic growth patterns that mirror the self-assembly of clusters^20^ by particle attachment (CPA)^21^, orientated attachment (OA) and selective hydrocarbon chemistry^22^. Such mechanisms have proved to be important regulators in the arrest of amorphous precursor phases^23^ under confinement^24^ and delayed crystallization in restricted spaces^25^ increasing the time scale of polymorph conversion^26^. Another important example relates to the chemistry of pore^27^ formation synonymous with confined spaces capable of limiting heterogeneous nucleation as a function of pore size. The concept of ‘nucleation in restricted spaces’ may have important implications in biomimicry for bio-mechanistic pathways that exhibit dependency between nucleation events and the order of dimensionality of porous materials^27^. This emphasizes the important relationship between chemistry and biology at the low dimensional scale gaining recognition from earlier works^7^. Such studies provide an insightful perspective on the chemical nature of porous cavities and their use in altering energy barriers to nucleation. Overcoming unfavorable energy barriers synthetically opens up possibilities to higher ordered structures that are otherwise more primitive in design by conventional routes.

Biology makes effective use of catalytic surfaces in cationic and anionic environments, surface charge effects, selectivity and spatial periodicity, interfacial energies and polymers by self-assembly from template structures^28^ to generate passivating surface ligands, pre-clusters as molecular building blocks by CPA and OA in confined spaces to self-assemble low dimensional materials. Here, we designed an approach to gain better understanding of the basis of biomimicry at the TiO_2_ surface. We used this to approach for the rationale design of sub-nanometer TiO_2_ to improve its photodynamic properties. Using the space restriction around alkyl-rich phosphonium centered cation with a poorly coordinated dicyanmide anion, the potential biomimetic surface properties of the titanium isopropoxide bulk precursor at the TiO_2_-ionic liquid (IL) interface was investigated. The periodically spaced mixed anionic–cationic IL enriched by phosphonium and nitrogen complexes tethered to long hydrocarbon chains formed the basis of a synthetic mimic rationalized to function as the desired biomimetic matrix. Here, the complementary interplay at the TiO_2_-IL interface driving enzyme-like bonding configurations at Ti and defect sites mirrors biogenic pre-crystalline growth^29^ exhibiting both shape and size tunability between metastable (amorphous), semi-crystalline and crystalline states. Solvation resulted in a self-assembled polystyrene–porphyrin–TiO_2_ (PS-P- TiO_2_) caged complex dominated by nitrogen^8,30^ and phosphine ligand chemistry. We further demonstrate that polymer compartmentalization of TiO_2_ is intrinsically coupled to band gap excitonic emission. Stabilized nanocaged TiO_2_ revealed an overall cell efficiency performance of 8.39% which suggests an increase in overall of charge transfer and cell efficiency by 2.85% and 0.54% *respectively* as compared to TiO_2_ alone.

## Templating Polymorph Selection at The TiO_2_-Polymer Interface Via an Amorphous Phase at The Quantum Scale

We investigated the biological relevance of a synthetic template as a potential biomimetic intermediary and chemical facilitator for the assembly of sub-nanometer TiO_2_ particles by confinement. We reasoned this to be possible using an alkylated phosphonium dicyanamide ionic liquid (IL) reaction medium composed of loosely ordered cationic-anionic ions. The possibility of inducing ‘slowed’ nucleation growth by nitrogen and phosphinic complexation with the catalytic TiO_2_ bulk precursor surface by self-assembly and the potential for polymer confinement by adsorption of hydrocarbon chains at metal oxide sites was explored as an alternative to using polymer additives. We sought evidence for the possible growth arrest of TiO_2_ to the quantum range at the polymer-metal interface by rapid positioning of charged monomer units polymerized within confined volumes^31^.

**Figure 1:**
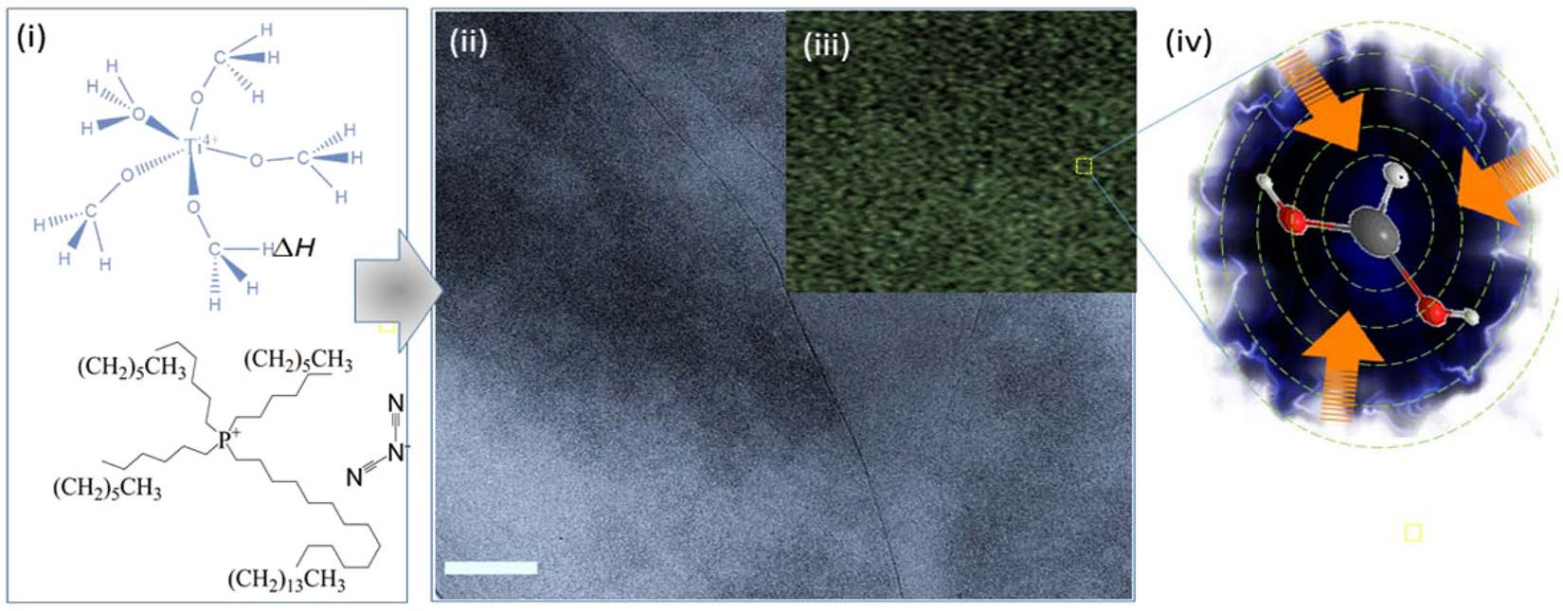
Synthetic stabilization of an amorphous network of TiO_2_ quantum dots (QDs) in an ionic liquid (IL) (alkyl phosphonium dicyanamide) reaction medium thermally driven at 120 °C. (i) Bulk titanium oxide and ionic liquid (phosphonium dicyanamide) precursor chemistry was used as an effective biomimetic reaction medium driving a (ii) densely populated assembly of TiO_2_ amorphous clusters of quantum trapped disordered aggregates by bright-field electron imaging. The inset (iii) shows an enlarged view of homogeneously sized clustered QDs under (iv) polymer confinement. The scale bar in (ii) is 100 nm.

**Figure 2:**
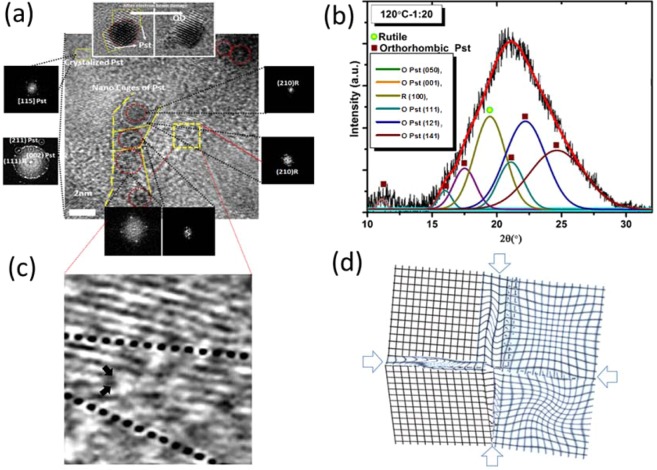
HRTEM and WAXS analysis of nanocaged TiO_2_ quantum dots (QDs) in polystyrene (PS). (**a**) HRTEM imaging of nanocaged of TiO_2_ QDs at the metal oxide and polystyrene interface. The nanocages are shown within the yellow borders typically in the size range of 1.25–2.81 nm (**b**) Deconvoluted WAXS pattern of polystyrene nanocaged TiO_2_ QDs in 1:20 fold ethanol exhibiting a crystal syndiotactic phase of the polymer and orthorhombic rutile TiO_2_ phase. (**c**) Dislocations at the boundary interface of crystal growth often lead to (**d**) deformities to reduce surface energy.

This hypothesis was supported by the formation of localized‘ growth patterns of TiO_2_ quantum dots (QDs) in a confined reaction space. Figure 1(ii) shows an ‘interaggregated’ amorphous network of non-crystalline spherical morphologies of semi-conductor particles at 120 °C assembled in IL medium with the bulk TiO_2_ precursor Fig. 1(i). The bright-field electron imaging in Fig. 1(ii) shows a high density of adjacently positioned nuclei seeds revealing unexpectedly, a high degree of self-driven organization at the sub-nanometer level which may have structural relevance to a pre-crystallization amorphous state in biomineralization. To explore the morphological significance and overlapping features with biologically induced growth mechanisms, HRTEM and WAXS analysis was used to determine the nature of the underlying growth heterogeneity at the boundary edge affecting size and shape flexibility of QDs. The striking monomodal size distribution of growth restricted nuclei suggests a ‘scaffold’ assisted growth pattern indicative of extensive polymer networks. Such adjoining networks have been shown to direct the arrest of non-crystallographic thin films and fibre-like morphologies of calcium carbonate using charge specific synthetic polymers^32^. In ethanol the presence of a co-polymer blend at the TiO_2_ interface is revealed by imaging of the periodic lattice fringes in the inset of Fig. 2a observed prior to electron damage (as shown within the yellow boundary). The analysis shows that the geometric spacing at the boundary is consistent with the polymer outgrowth of polystyrene (denoted here as Pst and elsewhere as PS). This observation was also confirmed by X-ray diffraction from the WAXS analysis (Fig. 2b) which also revealed a distinguishable blending polymer phase corresponding to (PSt) which conformed to a rhombohedra type geometry with hexagonal axis lattice parameters; a = b = 2.12288 and c = 0.65 nm (space group $R\bar{3}c$). This conformation is formed at the boundary of the tetragonal phase of rutile TiO_2_ with lattice parameters a = 0.46047 and b = 0.2909 nm (Table S1). The confined size growth of TiO_2_ in the sub-nanometer range of 1.25–2.81 nm shown by the TEM image in Fig. 1(ii and iii) agrees well with WAXS analysis (Fig. 2b). Table S1 shows that increasing the content of solvent from 1: 10 to 1: 20 (v/v) enhances the crystallinity of Pst but decreases the polymer pore size. We assert that the process describes a new order of precision that parallels naturally occurring biological interactions. By analogy, self-complementarity among structural components begins from evolving monomers that are dynamic and adaptive to a changing nucleating surface triggering anisotropic behaviour. In fact, the anisotropic properties of the polymer are affected both by temperature and solvent. Table S1 shows that polymer tacticity of polystyrene alters the crystal symmetry with a change in temperature from isotactic to syndiotactic. Thus, the ‘nanocaging’ of TiO_2_ Fig. 2a provides evidence that the geometrical nucleating surface of polystyrene is actively responsive to the changing face of the semiconductor surface resulting in growth arrest. The functional biomimcry of polystyrene caged TiO_2_ is comparable to lipid-protein trapped vesicles of iron oxide^33^ that exert stress and strain at the boundary of contrasting interfaces. The overlapping polymer and TiO_2_ crystallographic planes and line defect at the boundary edge are shown in Fig. 2a,c respectively. Such imperfections and deformities (Fig. 2d) are likely to form during the early stages of growth and here, the pre-assembly of amorphous networks signify the dominance of rapidly driven polymerization kinetics around growth restricted TiO_2_ nuclei. However, crystallization from the amorphous state is easily achieved in ethanol suggesting that solvation plays a minor role in the ordering of the crystallographic phase by lowering the surface energy. Thus, sizeable reductions in interfacial energies at the intersection of soft-hard surfaces such as metals and polymers can introduce new relationships between nucleating geometries, size and confinement in a metastable configuration.

## Configuring Functional Biomimicry at The TiO_2_ Surface: Supramolecular Compartmentalization of Polystyrene (PS) Caged TiO_2_ Bridged Via Porphyrin (P) Rings in The Self-Assembly of PS-P-TiO_2_

### Deciphering the fundamental building blocks of PS-P-TiO_2_ at the IL-TiO_2_ interface

**Figure 3:**
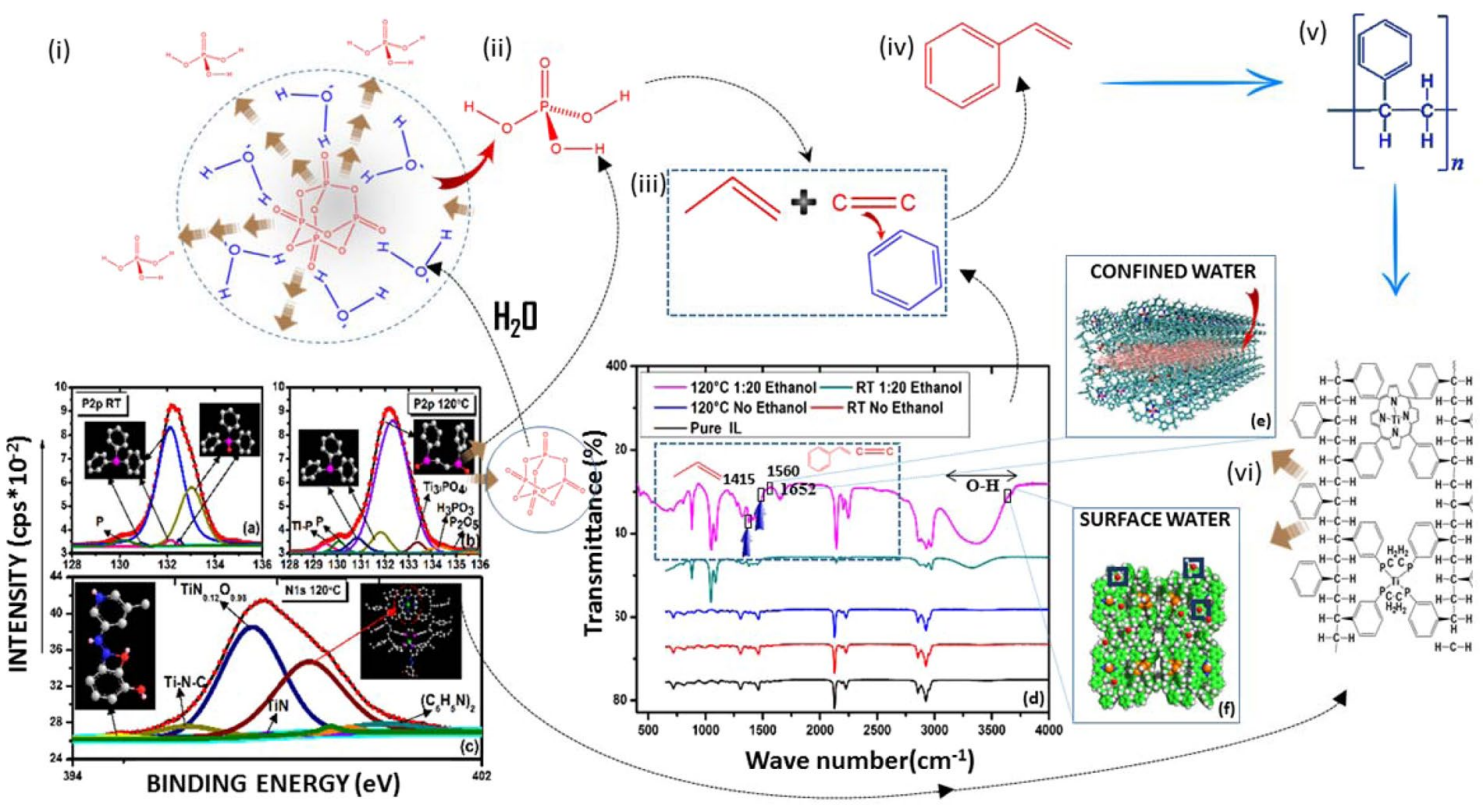
Key chemical steps in the quantum scale polymer confinement of TiO_2_. (i) The hydrolysis of phosphorous pentaoxide (P_2_O_5_) is evidenced by (**b**) P2p X-ray photoelectron spectra (XPS) (BE 135.596 eV) leading to the formation of (ii) phosphoric acid (H_3_PO_3_) (P2p; BE 133.78 eV). This was supported by surface analysis using XPS (shown in **a**–**c**) and FT-IR (shown in **d**). FT-IR analysis signifies band vibrations corresponding to (iii) vinyl and C=C stretches around 1415 and 1560 cm^-1^ respectively. The formation of (iv) styrene monomers that polymerise to (v) polystyrene forming a scaffold around TiO_2_ bound porphyrin rings (C36H46N4) (N1s; BE 398.73 eV) signified by Ti-N ligand chemistry (N1s; BE 398.18, 397.59, 396.7 eV) shown both (vi) schematically and by computational modelling in (**e**). In the FTIR spectrum shown in (**d**), the hydration state of PS-P-TiO_2_ is reflected by water confinement (shown in (**e**)) in nano or sub-nanometer polymer pores with bond vibrations at 1652 cm^-^1 and the bulk state of water reflected by OH vibrational states around the broader peak (3700–3100 cm^-1^).

The concept of segregated growth and bio-driven compartmentalization has important biological relevance in the co-assembly of materials with incompatible surfaces. We sought evidence for amorphous driven compartmentalisation at the catalytic TiO_2_ surface through bond activation of C─C, C─H, C─N C─P and O-Ti─N. The functional biomimcry of compartmentalisation is a distinguishable feature permitting undesirable polymer chain growth around metal oxide nucleation sites. We used X-ray photoelectron spectroscopy (XPS) and Fourier-transform infrared spectroscopy (FT-IR) to map the cooperative conformational use of space under strained geometries, charge, functional group selectivity, particle assembly and alignment of interacting particles at surfaces and sub-surfaces to establish the structural composition at the polymer-organometallic interface. In ethanol, the amorphous to a crystalline phase transition of the metal-organic assembly was broadly surface sensitive to the binding energy (BEs) peak at 284.5 eV in the C1s spectra (Table S2). This suggests the presence of an extensive carbon-hydrogen structural scaffold and signifies ‘out-of-plane’ C=C vibrational bands (1494–1453 cm^−1^) that are often associated with styrene complexation^34^. The detection of residual quantities (<2%) of phosphorous penta-oxide (P_2_O_5_) with binding energy (BE) of 135.6 eV (Table S2) in the P2p XPS spectra (Fig. 3b) may be important to the availability of phosphoric acid (H_3_PO_3_) (BE of 133.78 eV; P2p) (Fig. 3b). Figure 3ii shows that it may have been liberated from its reaction with water. Depleted levels of the acid precursor suggest that phosphoric acid may play an important role as an initiator in crosslinking of styrene monomers to form polystyrene chains via its interaction with vinyl and ring aromatic groups such as benzene (Fig. 3iii). The C=C bond stretch around 1494–1453 cm^−1^ (Fig. 3d) likely originates from the self-assembly of benzene and styrene molecules as precursors to polystyrene (PS) assembly (Fig. 3i–iv) leading to polystyrene (Fig. 3(v)).

**Figure 4:**
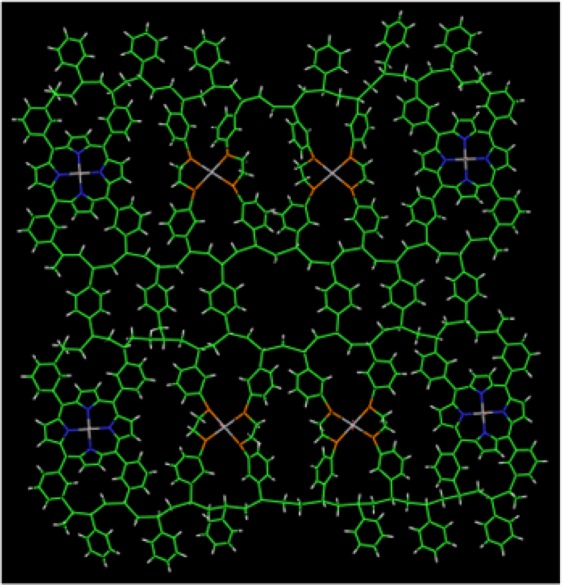
Modelled structure of 1-dimensional PS-P-TiO_2_. Computational modelling of 1-dimensional projection of quantum confined porphyrin compartmentalised TiO_2_ embedded in a polystyrene polymer lattice. Modelling colour scheme; white is hydrogen; cyan is carbon, red is oxygen, blue is nitrogen, gold is phosphorous and pink is Ti. The structure was built using Discovery studio (ref: Dassault Systèmes  BIOVIA, Discovery Studio Modeling Environment, Release 2017, San  Diego: Dassault Systèmes, 2016).

Further, weak vibrations associated with a pool of vinyl groups emerging at 1415 cm^−1^ in the FT-IR spectrum (dashed box in (Fig. 3d)) could potentially behave as an effective PS crosslinking agent^35^. Such polymerization events have much relevance to the quantum confinement of TiO_2_. This is supported by nucleation mechanisms in which semi-crystalline intercalation at metal-polymer hybrid interfaces have been shown to assemble with polyethylene-block-polystyrene (PE-b-PS) into segregated nano-domains effectively causing metal growth arrest within the PS matrix^36^. A change in the packing structure of this order is also reflected in the comparative FT-IR profiles (Fig. 3d). Further, analysis by XPS reveals substantial modification of the bulk TiO_2_ surface generating template induced oxygen deficient vacancy sites around Ti^3+^ residues. This is reflected by an asymmetric Ti2p(2p^3/2^) peak spectral shape corresponding to BEs 456.25, 457.60 and 461.96 eV (Table S2 and Fig. S1). The accompaniment of a complex spectral pattern of the bond order TiN_0.12_O_0.98_ (BE N1s; 397.59 eV) that correlates to a porphyrin ring arrangement of the N1s peak with BE 398.97 eV suggests that Ti^3+^ passivation occurs via sub-surface Ti–N ring compartmentalisation (Fig. 3c and Table S2). This leads to the assertion that the growth confinement of TiO_2_ is chemically locked in a 4 N or 2 N porphyrin ring geometric arrangement embedded in the PS scaffold. Strong orbital overlap between Ti and two oxygen atoms shown in the spectral for O1s with BEs; 529.71, 530.34, 531.62, 531.95 and 533.30 (Table S2) is consistent with the nucleation and growth of TiO_2_. We infer that quantum scale confinement of TiO_2_ is enthalpically driven by Ti–N bond formation that may kinetically exceed porphyrin and polystyrene polymerization at temperatures below the glass transition state of polymer crystallization. This scenario predicts the formation of a carbon-hydrogen-nitrogen polymer scaffold arresting TiO_2_ nucleation via Ti–N bond associations in nano-size cavities. We propose that sub-fractionation of polymer growth induces a ‘cage-within-a-cage’ architecture comprising nanometer sized PS crystals causing the quantum confinement of TiO_2_ confined in periodically bridged porphyrin molecules. This hierarchal assembly evidenced by the association of aligned phenyl moieties of polystyrene (BE C1s; 284 eV) with porphyrin rings (BE N1s peak; 398.73 eV) (Fig. 3c and Table S2) is shown in Fig. 4. Entrapment of TiO_2_ at the polystyrene-porphyrin boundary suggests that nitrogen doping of Ti_2_O_3_ (Tables S2 and S3) via Ti–N bond activation is an effective route for nanocaging the metal oxide within the PS-P matrix.

In the presence of ethanol, the appearance of vibrational frequencies around 1096 cm^−1^ and 3300–3500 cm^−1^ (Fig. 3d) in PS-P-TiO_2_ also signifies the dominant role of nitrogen chemistry in the formation C–N and N–H bonds respectively (Table S3) at the TiO_2_ surface. This provides supporting experimental evidence for the dissociation of nitrogen from chemisorbed nitrogen complexes (IL) and validation for theoretical calculations that favour low activation barriers for nitrogen dissociation on rutile surfaces^37^. A probable route to nitrogen reduction to NH from nitrogen-rich anions at the TiO_2_ surface could occur by direct N–N bond scission via bridging oxygen (O-br) vacancy active sites on rutile (110)^38^ implying a critical role for O-br defects in nitrogen chemistry. However, the dissociation of C–N and N–H bonds from surface adsorbed dicyanamide stabilized by water molecules adsorbed onto bridge-bonded oxygen vacancies might facilitate an alternative route to incorporate nitrogen as a building block for the assembly of complex functional structures as evidenced here. The findings indicate as reported elsewhere^39^ that the TiO_2_ surface is more selective towards associative pathways for nitrogen reduction.

### Orientated particle attachment as a route to biomimcry in the assembly of PS-P-TiO_2_

**Figure 5:**
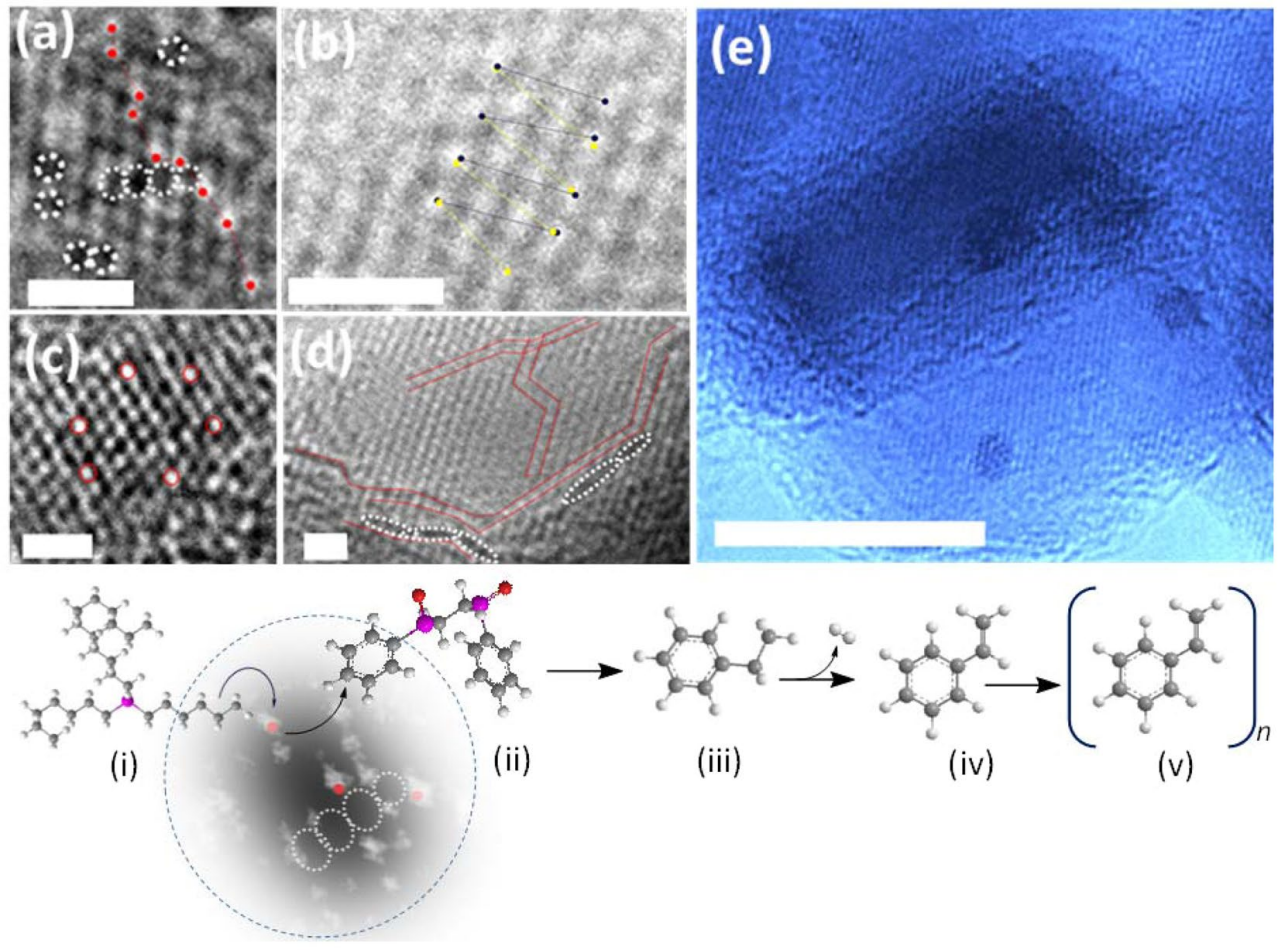
High resolution TEM imaging of defect states in quantum confined TiO_2_ in PS-P-TiO_2_. The images show the intensity modulation of atomic-sized images from HRTEM. Larger atoms (e.g. P atoms) are visibly brighter and broader in comparison to other regions in the lattice. The red closed dots in (**a**) show a ‘step-edge’ like behaviour of surface complexed phosphine ligands occupying TiO_2_ oxygen deficient point defect sites encircled in white and shown as dark regions in (**b**). The image in (**c**) shows substitutional point defects by larger atoms. In (**d**), polymer assembly is shown to adopt a ‘slip’ growth mechanism which appear to form screw dislocations in (**b**). The pattern follows the route shown within the red bordered lines along vacancy defect sites encircled in white. The colored HRTEM image in (**e**) shows more clearly the ‘slip’ growth layered architecture. The chemical Scheme in the lower panel represents a possible convergent route with Scheme 1 in Fig 3. The interaction of (i) alkylated phosphonium molecules at defect sites (red closed circles) results in catalytically driven (ii) aromatization of linear alkyl chains. (iii) Surface decomposition and dehydrogenation of ethyl benzene from 1,2-ethanediyl)diphenylphosphorane oxide (P2p; BE 132.3 eV) selectively forms (iv) styrene monomers (C1s; BE 284.5 eV) polymerizing into (v) polystyrene networks in the presence of H3PO4 (P2p; BE 134.32 eV). All scale bars are 1 nm.

In nature, the physical shape, size and composition of interacting substrates with enzymes for example, determine if bonds can be broken and formed upon binding by overcoming barriers to activation energies. Innovative experimental approaches have shown that the catalytic nature of metals and their oxides is strongly dependent on size, shape and composition^40^. In fact, catalytic selectivity at the nanoscale is drastically altered^41^. Oriented assembly occurs by spontaneous particle fusion along a common growth axis and rotation at interfacial boundaries^42^. This is accompanied by bond formation which serves to reduce surface energies at interfaces. In PS-P-TiO_2_, mismatch growth patterns (Fig. 2c) were also accompanied by anti-phase boundaries forming atomically spaced ‘step-like’ layers of polymer along the same growth axis and showed hallmarks of rotational fusion at the edges (Fig. S5). In the absence of chemical additives such as surface binding ligands that passivate high energy dangling bonds during nucleation^14,15^, we assert that programmable nucleation growth of TiO_2_ is better orchestrated by energy changes that occur congruently with the evolving surface from a chemically inert to a functionally active arrangement. This implies an alternative mechanism of hierarchical growth that occurs through the formation of surface-assembled particles or clusters different to atom-by-atom growth. Defects states such as vacancies, interstitials, edges, curvature and anti-phase boundaries (Fig. 5a) result from the miniaturization of particles from the bulk to sub-nanometer dimensions at the metal oxide surface. The morphological contours of defect sites behave as active sites for interacting particles that can effectively bind through adsorption processes. Intermediate products that result from the introduction of new chemical functionalities through catalytic transformations embody the structural framework that mirror the changing energy configurations of defect states as they evolve by biomimetic strategies (Fig. S6). This is coupled to activation barriers intrinsic to the defect surfaces permitting the dynamics of site-specific cluster formation from the bulk reaction medium on the time scale of heterogeneous nucleation. The demand for precision in structural design of metal organic frameworks (MOFs) require a better understanding of energy supply to trigger reaction pathways of interest. In the ‘assembly of PS-P-TiO_2_, some clues to the nature and composition of intermediate pre-clusters and clusters have been identified using the structural methods described in Figs. 2 and 3. In orientated attachment, local disorder at defect sites play a role in particle alignment of pre-clusters and clusters, directing their movement (*e*.*g*. rotational motion) and co-alignment while adopting growth patterns to minimize energies at interfaces. Analysis by XPS (Fig. 3c and Table S1) shows that chemisorbed dissociation of nitrogen-rich complexes at the TiO_2_ surface induces oxygen vacancy sites (Ti^3+^) largely altering the charge distribution. The replacement of N with O which is compensated by Ti–N bond formation and the occupation of N at interstitial positions also provides opportunities for the formation of passivating ligands (Table S2). The relationship between crystal size and shape and their correlation with surface properties is summarized in (Table S1). Analysis shows that the Miller indices of rutile are preferentially associated with the (100) and (210) type facets and correlate well with growth dynamics along the plane of uncoordinated sites at the reactive surface with changing solvent concentration. This suggests that oxygen vacancy sites likely to play a key role in the precursor decomposition of nitrogen-rich dicyanamide molecules releasing nitrogen precursors, C≡N and C─N. Reportedly, an important observation is the aromatization of linear hydrocarbon alkane chains via C─H bond arrangement. Selective chemisorption of pre-clusters in the direction (100) and (210) reveals strong relevance to electronic fluctuations in Pt^43^ along (100) and (210) accompanied by the release of H_2_, CO^44^ and reactive radicals as important intermediates of metal induced catalytic reactions. Such a mechanism might have relevance in the stabilization of Ti defect sites via Ti─N bond formation and the restructuring of phosphine derived alkane chains around Ti─N centers into a porphyrin configuration. Tables S1 and S2 however, provide some insight on the intrinsic nature of the planes and their dominance in Ti─N bond stabilization. From Table S2, it is evident that porphyrin assembly associated with Ti_2_O_3_ defect sites at the TiO_2_ surface shows small variation in the area percentage change from RT to 120 °C. For example, a small change in the area percentage of Ti_2_O_3_ (2p3/2) with temperature variation BE’s around 457.65 and 456 0.89 eV) (Table S2) indicates that defects are atomically passivated by nitrogen surface capping. Table S1 however shows that organic and organometallic adsorbates are less tightly associated to defect sites along the R(100) direction signified by a change in the lattice parameters (TiO_2_). Here, a change in the solvent environment increases surface mobility favoring the re-orientation of molecules to lower surface energies. In contrast, strong immobilization of porphyrin via Ti─N coordination imposes a ‘capping’ effect on the (210) plane stabilizing the surface. The surface dynamics along ‘h’ direction provides for greater heterogeneity in catalytically driven reactions at reactive defect sites.. The atomic reconstruction within oxygen deficient sites (dotted white bordered dark regions shown in Fig. 7(a,d) provides an important mechanistic clue for the involvement of alkylated P atoms in polymer growth around porphyrin entrapped TiO_2_. The direct visualization of atom migration was observed atomically using resolved HRTEM. Here, Frenkel defect pairs O_I_ (red dots) and V_I_ (dotted white circle) shown in Fig. 7 (a,d) results from both oxygen ion mobility and substitutional point defects originating possibly from the larger atom migration (Fig. 7c; ‘bright spotted’ P atoms encircled in red). Further, the imperfectly oriented attachment of atoms lead to screw (Fig. 7b) and edge (Fig. 7d,e) dislocations. These defects direct the formation of ‘step-edge’ and ‘slip’ layered type architectural growth. The slip growth architecture shown in Fig. 7(d,e) is indicative of weak binding which supports flexibility at the metal-organic interface. The elimination of defects correlates well with the alignment of P atoms with along Ti^3+^ defect sites. However, metal-ligand interaction results in site specific aromatization of linear alkyl chains at P attachment sites to tri-phenyl phosphine oxide (TPPO) (P2p; BE 132.5 eV) (Table S2) at RT. Temperature induced growth at 120 °C leads to accelerated growth modification of TPPO to 1,2-ethanediyl)diphenylphosphorane oxide (P2p; BE 132.3 eV) (Table S2) accompanied by a substantial increase in the area % from 0.5 to 75.4 respectively. The infinitesimal shift in the BE between phosphine and phosphorane indicate that clusters chemisorbed around Ti^3+^ defects retain structural flexibility at moderate temperatures in solvent. The ease in structural conversion is favoured by a delocalized state increasing surface stability due aromaticity of alkyl chains. Catalytic selectivity and weak dissociative binding along defect sites has important implications in pre-cluster assembly. Like chemical reactions that occur under bio-compartmentalization in shape changing environments, the migration and evolution of larger self-assembled clusters at defect interfacial boundaries are perhaps complementary to rates and shape transformations^45^ at chemisorbed sites. While reaction rate and selectivity is controlled by size and shape on metallic nanoparticles^46^, the Scheme in Fig. 5 shows that catalytic selectivity at the surface is a tunable entity for an evolving cluster and is intrinsically coupled to alterations in chemical functionality via bond activation^47^. XPS confirms the HRTEM observation and shows temperature induced changes in defect behaviour from RT to 120 °C favoring dramatic surface reconstruction. This is mapped by the decomposition of 1,2-ethanediyl)diphenylphosphorane oxide (P2p; BE 132.3 eV and Fig. 5; Scheme ii). The dissociative loss of aromatized phosphine oxide P=O functionality is marked by changes in bond activation to C=C (Fig. 5; Scheme iv) with the release of styrene monomers (Table S2; C1s; BE 284.5 eV) by dehydrogenation of the possible intermediate structure; ethyl benzene (Fig. 5; Scheme iii). Hence, controlling the metamorphosis of shape dynamics of ‘multi-pathway’ defect-cluster interactions holds the potential to release unique geometrical structures via surface energy driven bond activation as observed for PS-P-TiO_2_.

## Confinement Uses a ‘Strained-Induced’ Growth Mechanism to Biomimic The Amorphous ‘Arrest’ of Quantum Caged TiO_2_

**Figure 6:**
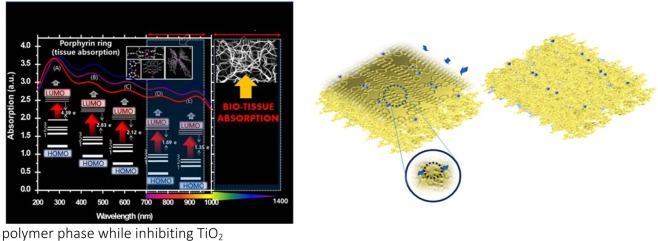
Optical profile of PS-P-TiO_2_. Spectral band shift of porphyrin (P) bound TiO_2_ in PS-P-TiO_2_ atypical of porphyrin ring characteristics signifying distortion of porphyrin ring compartmentalization of TiO_2_ via Ti–N bonds within the ring cavity. This shift occurs from 290 nm to 286 nm influencing the excitation properties of the neighbouring Soret band. Stabilization of the strained porphyrin–TiO_2_ complexation (adjacent figure) supported by the surrounding polystyrene (PS) polymer lattice layer via the (1) aromatic moiety groups of PS and (2) phosphine-derived surface ligands provides the opportunity to increase the therapeutic window of TiO_2_ for tissue absorption at the quantum scale. Copyright Wiley-VCH Verlag GmbH & Co. KGaA. Reproduced with permission.

It is not unusual for living systems to ‘switch’ from a precursor amorphous phase to a crystalline state to synchronize and adapt to environments based on size and shape morphology. Transient amorphous CaCO_3_^48^ and biopolymers^49^ are examples of regulated networks in which a readily ‘distinguishable’ amorphous matrix coexists within regions of crystallinity. In the absence of solvent, high resolution TEM of PS-P-TiO_2_ (Fig. 1) show growth restricted crystals of TiO_2_ in confined spaces in an amorphous polymer network. This suggests that polystyrene polymer uses short range order to adapt to the size and shape of surrounding crystalline metal centers. We sought evidence for biomimicry in the growth arrest of TiO_2_ at the polymer-metal interface from the rapid positioning of charged monomer units polymerized within confined volumes^31^. We previously discussed the effects of distortion around the porphyrin-TiO_2_ complex^8^ and here we aim to discuss how polymer blends at the metal interface might experience constrained growth and interfacial stresses. The transition from a periodically spaced environment from the IL bulk state to ring confinement relies on bond distortion to minimize the interfacial surface energy during heterogeneous nucleation. We infer that the restricted growth characteristics of TiO_2_ coupled with size and shape selectively is mainly affected by the binding symmetry at the metalloporhyrin site manifested by C─N, and Ti─N bond ring deformity in accommodating TiO_2_ which is evidenced by the large spectral red shift of the Soret (B-band) from 422.1 to 446.1 nm observed in the abs-λ profile (Fig. 6). The peak shift also signifies the presence of PS as an anchoring framework to porphyrin and distortion of the phenyl moiety at the P-PS boundary where the alignment is affected by positioning of TiO_2_ at the porphyrin center. This scenario correlates well with a rapidly evolving amorphous phase at the out-growth of polymerization over a slower ‘ordered’ phase^50^. This phenomena is the cornerstone to interfacial stresses at the metal-polymer boundary where growth restraints dominate over particle size. Thus, predictably surface energies favour non-crystallinity of an amorphous polymer phase while inhibiting TiO_2_. nucleation in low-dimensional porous cages. Two melting peaks in the differential scanning calorimetry (DSC) analysis (Fig. S2) observed for PS complexation shows the co-existence of a high and low crystalline form corresponding to the MOF configuration and a semi-crystalline state forming at 408 and 41 °C respectively that resides well above and below the glass transition temperature (T_g_) of the polymer around 100 °C for the uncomplexed PS. This may arise due to fractionated polymerization. Polymer confinement to nanometer scales and the associated changes in transition glass dynamics further reflect the cooperative thermal motion and confined geometries intrinsic to the polymer blend. The dissimilarity with protein behaviour however is marked by an anomalous trend exhibited by a strong-to-fragile transition evidenced by a high-to-low fragility index (T_m_/T_g_) T of the quantumized TiO_2_ caged polymer blend upon gradual cooling under ambient conditions. Low temperature glass transition dynamics of proteins evidenced by the ‘fragile-to-strong’ shift in the behaviour of associated water molecules^51^ has recently been demonstrated for cerium oxide^52^. The suppression^53^ or enhancement^54^ of protein glass transition dynamics mediated by strongly interacting chemical environments that include solvents and biopolymers suggests that the phenomena of synthetic polymer confinement self-assembled via a templated matrix may also obey similar behaviour glass-like characteristics across a material dependent temperature range. Table S4 compares the order of difference in the T_g_ of polystyrene in PS-P-TiO_2_ with the bulk states at 120 °C and RT respectively revealing drastic changes in polymer properties with the bulk characteristics. While the fragility index provides a good indication of stress-relaxation behaviour^55^, transition of PS to the fragile state in crystalline PS-P-TiO_2_ (in ethanol) from an otherwise ‘strong’ PS configuration of the bulk state (Table S4), mirrors the ‘plasticized’ effect of variable water mobility under confinement of biopolymers^56^. We have discussed previously the tacticity of PS-P-TiO_2_^8^ a structural regulator in PS likely plays a prominent role in bond selectivity permitting crosslinking molecules and bridging structures. This allows the construction of a supramolecular PS matrix transitioning from an amorphous, semi-crystalline to the crystalline phase triggered by solvent. Hence, polymer-metal amorphous states achieved at moderate temperatures within the close range of the glass transition state of self-assembled polymer materials undergo heterogeneous nucleation to glassy colloid structures. Such disordered patterns can ‘switch’ to a crystalline state through stimuli-triggered environments.

## Solvent-Water Dependent Polymorphism of Surface Modified TiO_2_ Typifying Biogenic Amorphous Character

Recently, a study by Gorshunov *et al*.^57^ revealed that the restriction of water molecules spatially inside nanocavities and their subsequent association with cavity walls exhibit properties that are directionally dependent and influence vibrational states and dynamic behaviour. Further, unusually bonded water networks can operate as templates inducing metal nanoparticles to adopt different shape directed geometries^58^. This arrangement is quite different to the mobility and diffusivity of water molecules in TiO_2._ Evidence that supports the dual orientation of water under conditions of confinement that result from polymer stress and strain is readily visible from the FTIR spectral bands corresponding to water binding states. The regional band vibration stretch at 3550 cm^−1^ (Figs. 3d and S3) of the broader band between 3000 and 3500 cm^−1^ (Figs. 3d and S3) is characteristic of the O-H stretch of water and is distinguished from the hydroxyl moiety of alcohol by the peak position at 1652 cm^−1^ (Figs. 3d and S3) synonymic with the scissoring frequency of the O-H pair of water. This might originate from ethylene oxidation releasing carbon dioxide and carbonic acid (Fig. S3(b); FTIR peaks at 800 and 1048 cm^−1^) as secondary products of olefinic chemistry. The dramatic blue shift signifies the retainment of water at much higher levels in the polymer organization at 120 °C different to the aqueous structuring around the polymer at RT in which the vibrational frequency at 1652 cm^−1^ is absent. The blue shift in the vibrational state of water to 1652 cm^−1^ may be correlated to increased hydration with greater flexibility in the rotational freedom of water to the more rigid confinement at the low frequency end at 3550 cm^−1^ (Figs. 3d and S3). Differences due to the packing of hydrogen bonded water molecules in mobile and constrained environments during self-assembly is profiled by differential scanning calorimetry (DSC) analysis as a result of temperature and solvation effects (Fig. S4). Here, binding preferences may be predominately lattice or coordinated^59^ and the thermal behavioral sigmoidal pattern in the presence of ethanol at 120 °C shows 56% loss of water occurs at 68 °C from the steep decomposition in (Fig. S4(g)). Under different conditions, water complexation and orientation in PS-P-TiO_2_ is demonstrably more rigid even in the presence of ethanol at RT and under different conditions (Fig. S4(d–f)).

**Figure 7:**
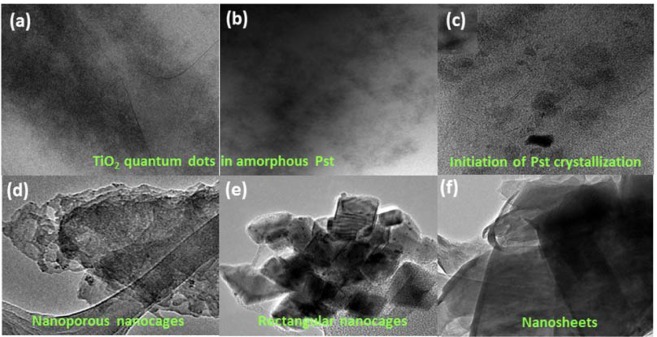
Solvent dependent morphologies of PS-P-TiO_2_. TEM bright field imaging indicating QDs amorphous matrix of polystyrene in (**a**) pure reaction (**b**) 1:5 (v/v) ethanol. (**c**) Initiation of crystallization of polymer indicated by the faceted weak contrast rectangular crystals of (~10–20 nm). The dark contrast is possibly due to entrapment of ionic liquid (**d**) Nanoporous cages (pore size-3–5 nm) at 1:20 (v/v) ethanol, (**e**) rectangular nanocages (15–50 nm) 1:40 ethanol (v/v) (**f**) Sheet like nanocages (500 nm–1 μm) diluted 1:60 (v/v) ethanol. Scale bars in images (**a**–**d**,**f**) is 100 nm and 50 nm in image (**e**).

**Figure 8:**
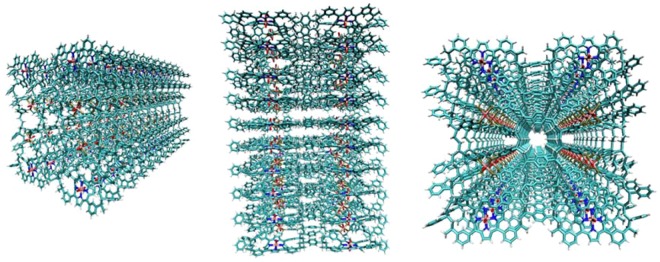
Computational modelling of 3-dimensional supramolecular PS-P-TiO_2_. The structure was built using Discovery studio (ref: Dassault Systèmes BIOVIA, Discovery Studio Modelling Environment, Release 2017, San Diego: Dassault Systèmes, 2016.) as single porphyrin units, and geometry optimised before combining to create the complete structure (consisting of eight units), which was then further minimised.  Structures were minimised using the MMFF94 force field. This monomer was copied to create 10 layers minimizing the system between each addition. Modelling colour scheme; white is hydrogen; cyan is carbon, red is oxygen, blue is nitrogen, gold is phosphorous and pink is Ti.

A colloidal suspension of the amorphous material in ethanol showed shape dependency on solvent concentration that progressed from the initiation of the crystallized state of a metal organic framework (MOF) in 10-fold excess of ethanol [Figs. 2c and 7a; 1: 10 (v/v)] to a crystalline nanoporous state under 20-fold excess of solvent [Fig. 7d; 1: 20 (v/v)]. The ease in the control of metal-polymer morphology was demonstrated further by varying solvent concentration generating rectangular nanocages and layered sheet structures in 40-fold [Fig. 7e; 1: 40 (v/v)] and 60-fold [Fig. 7f; 1: 60 (v/v)] ethanol-MOF reaction mixtures. The anisotropic behaviour between the inorganic and organic network is thought to be the result of phase separation which is being prevented by their attachment^60^. The SEM images (Fig. S8) of self-assembled polystyrene chains bear some similarity to grafted polystyrene on silica nanoparticles^60^ that self-assemble into a range of poly-dispersed super structures. The effects of solvent dependent anisotropy observed here is reminiscent of biomolecules in transition to more favorable conformations that make use of molecular associations and binding modes at the solvent─water─polymer interface to adopt energetically better orientated configurations through such chemical contacts. From a biomimetic perspective, by analogy, the energy landscape of a ‘folding funnel’ type trajectory depicted in Fig. S7 highlights the importance of controlling surface chemistry. Knowledge of the parametrics of catalytic defect chemistry will help aid the entrapment and stabilization of novel non-equilibrium structures in thermodynamic pockets via kinetic pathway selection *e*.*g*. mimicking metastable amorphous states. The existence of kinetic barriers that arise around glass transition state temperatures of polymers often prevent polymer crystallization to completion at the nanometer and sub-nanometer scales. Such intermediate structures are suggestive of high energy state structures that deviate from the lowest free energy states composed of uninterrupted adjoining chain lengths with highly ordered extended configurations. This occurs by taking advantage of the interchangeability between non-equilibrium states^61,62^. We have used computational modelling^63^ to show that supramolecular assemblies based on PS-P polymer confinement of TiO_2_ interconnected with bridging linkers (identified by XPS) are structurally and energetically feasible (Figs. 8 and S9).

## Application of Nanocaged PS-P-TiO_2_ QDs in The Efficiency Enhancement of Quantum Dot Dye Synthesized Solar Cells (QDDSSCs)

Recent interest in tunable quantum confined MOF supramolecular structures^64^ suggest that the structural periodicity governed by a nanoporous landscape embodies surface electronic states that are strongly seeded in the degree of dimensional confinement^65^. The UV spectral tail extending beyond the intensity observed at RT is consistent with the imaging of quantum sized particles by HRTEM (Fig. S10) and selective stabilization of diphenylphosphorane oxide and porphyrin ligands might originate from the establishment of narrow energy gaps increasing the orbital overlap. This is expected to facilitate donor-acceptor pairing between electron rich T─N bonding and delocalized ringed structures with electron deficient metal oxide d orbitals increasing the energy of HOMOs (highest occupied molecular orbitals) accompanied by decreased energy of LUMOs (lowest occupied molecular orbitals). This could be a strong factor in forming a luminescent metal-to-ligand charge-transfer (MLCT) excited state^66^.

**Figure 9:**
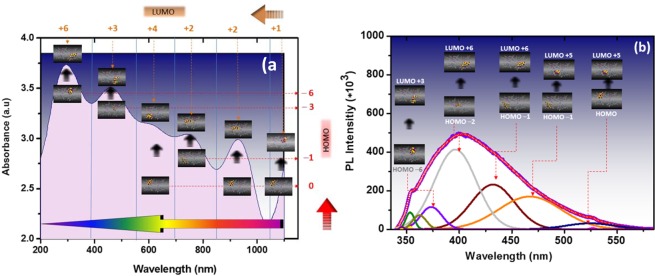
Correlation of wave-length with absorbance spectral data and photoluminescence measurements and modeled structures. Peak intensities of wavelength-abs characteristically occur along the Soret or B band transitions in the colloidal MOF assemblies that correlate to HOMO – LUMO arrangements along the conjugated structures. The peak maxima were converted to energy bands. Relative HOMO-LUMO positions for both (**a**) UV spectra and (**b**) photoluminescence intensity data were compared and asiisgned to modelled structures (based on experimental data). The comparison shows that there is good agreement in the energy jumps in the electron absorption excitability of the nanocaged structures.

Correlation of the force field stabilized complex with quantum wave functions of interacting molecular orbitals of existing interfacial boundaries was achieved by calculating the energies corresponding to the UV peak positions at wavelength λ (Fig. 9a) to computed HUMO – LUMO transitions obtained from model calculations (Fig S11). The spectrum range of the colloidal complex spanning the UV, visible and infra-red range was observed for photoluminescence (PL) peak positions corresponding to energies in the range of −1.16–4.33 eV (Table S5) which might to some extent satisfy the band-gap energy state requiring a reduced HOMO and LUMO gap between TiO_2_. In particular, the charge density distribution along the linker of LUMO + 2 (Fig. S11 and Table 5) and the metal charge distribution of HOMO/HOMO −1 are characteristic of elongated electron dense regions arising from bond stretching during HUMO-LUMO orbital overlap. These band energy assignments are of the same order deduced from the photoluminescence data in the range −2.3–3.5 eV (Table S5).

**Figure 10:**
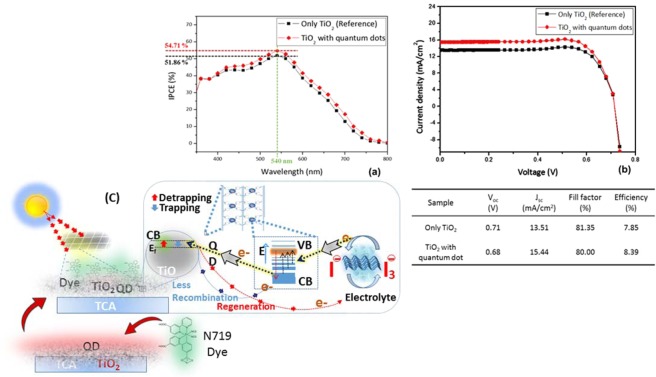
Solar cell performance and predicted mechanism of the QD based solar cell. (**a**) Incident photon to current efficiency (IPCE) of PS-P-TiO_2_ peaks at 2.48% relative to the reference cell at 540 nm (**b**) Current density–voltage curve and Table of parameters showing overall efficiency enhancement of 8.39% in QDDSSC performance (**c**) possible mechanism of trapping-detrapping route via dye (N719) adsorbed PS-P-TiO_2_ QDs.

To test DSSC performance, N179 dye was used as a co-sensitizer to enhance the photovoltaic performance of the newly fabricated PS-P-TiO_2_ assemblies for their depth of interaction with the embedded dye. However, the substantially poor absorption capacity of porphyrin in the Soret band range (0.02%) (Table S5) in the film was consistent with its expected poor photoluminescence characteristics. N719 dye was employed to counterbalance the sensitizing properties of porphyrin in complexed TiO_2_ with the possibility of improving better conjugation of the out-of-plane aromatic rings anchored to the central porphyrin core diminishing steric limitations. Figure S10 shows the morphological deposition of TiO_2_ QDs (indicated by the black arrows) as a HRTEM atomic level bright field image. The incident photon-to-current efficiency (IPCE) measurement in Fig. 10a reveals an enhancement of 54.71% relative to the reference cell (51.86%). This correlates well the overall of charge transfer and cell efficiency of 2.85% and 0.54% at a peak maximum of 540 nm (Fig. 10a,b) in the presence and absence of TiO_2_ QDs respectively. The enhanced photovoltaic cell performance correlates well with the photo-responsive behaviour of confined TiO_2_ quantum dots in which crystalline colloidal surface is optically active to absorb across the full spectrum of emitted photons (Fig. 10). Further, the detection of symmetric and asymmetric peaks of variable depth in the electron paramagnetic resonance (EPR) spectra (Fig. S12) is characteristic of the anisotropic nature of the spin state of free radicals and unpaired electrons of nuclei. The proposed mechanism is discussed in the Supplementary Material.

**Figure 11:**
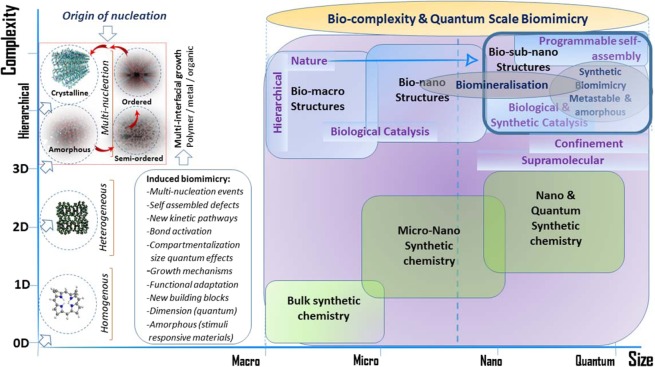
Biomimicry as a route to achieve quantum trapped hierarchical materials. Induced biomimcry in synthetic systems leads to a number of atomically important events (summarised in the closed box). Programmable self-assembly provides a feasible route at heterogenous interfaces for the quantum confinement of materials and tuning the complexiety of hierarchical structures as a function of size (boxed in the red border and and the blue border outlined in bold). This has important implications for conventional synthetic routes limited by kinetic pathwy accessability to higher structures.

The findings offer renewed interest in the rationale design of synthetic routes with the potential to trigger amorphous driven biomimetic pathways (Fig. 11) that have shown to exert quantum confinement effects for ‘hard-to-achieve’ organometallic architectures. Incorporating both structural and chemical determinants in processes important to biomimicry will ensure the availability of smarter, more efficient and functionally superior nanocontainer type^67^ materials.

## Experimental Section

Synthesis of Polymer nanocaged TiO_2_ Quantum dots have been synthesized by a one pot reduction process similar to the method previously reported by Khare *et al*.^68^.

### Characterizations

Wide-angle X-ray scattering (WAXS) and XRD were performed using CuKα radiation for 2θ ranging from −100° to + 168° (WAXS) and 10° to 85° (XRD). HRTEM imaging was performed by using JEM03013 (JeOL) with a resolution of 0.14 nm for lattice imaging and 0.17 nm for point imaging. X-ray photoelectron spectroscopic measurements were conducted on Kratos AXIS-Hsi spectrometer using a monochromatic Mg-K X-ray source fitted with a multi discrete detector system. UV- Visible measurements were conducted on Agilent Cary 60 UV-Vis Spectrophotometer and photoluminscence measurements were performed with Raman/PL instrument using 325 nm laser (20 mW), ND 100% filter, Range: 340~800 nm and Grating: 1,800.

### Modeling tools

Based on the experimental data, molecular modelled MM2 force field calculations (Cambridge soft chem3Dultra 10) was used for structure prediction substantiated by geometrical optimization calculations. Molecular orbital calculations employed the extended Huckel model.

## Supplementary information

Quantum scale biomimicry of low dimensional growth: An unusual complex amorphous precursor route to TiO2 band confinement by shape adaptive biopolymer-like flexibility for energy applications
