# Supplementary material for: Quantum scale biomimicry of low dimensional growth: An unusual complex amorphous precursor route to TiO2 band confinement by shape adaptive biopolymer-like flexibility for energy applications
Source: Sci Rep. 2019 Dec 10;9:18721. doi: 10.1038/s41598-019-55103-z (PMC6904763; doi:10.1038/s41598-019-55103-z)
Supplement: Supplementary file 1 — Quantum scale biomimicry of low dimensional growth: An unusual complex amorphous precursor route to TiO2 band confinement by shape adaptive biopolymer-like flexibility for energy applications [file 41598_2019_55103_MOESM1_ESM.pdf]

Supplementary Information

Quantum scale biomimicry of low dimensional growth: An unusual complex amorphous precursor route to TiO_2 band confinement by shape adaptive biopolymer-like flexibility for energy applications

Dahyun Choi[1#], Sanjiv Sonkaria[2#], Stephen J Fox[3], Shivraj Poudel[4], Sung-yong Kim[4], Suhee Kang[1], Seheon Kim[4], Chandra Verma[3], Sung Hoon Ahn[†2,4], Caroline Sunyong Lee[Ŧ1], and Varsha Khare[1*]

[#]These two authors contributed equally to this work

Tables

Figure S1

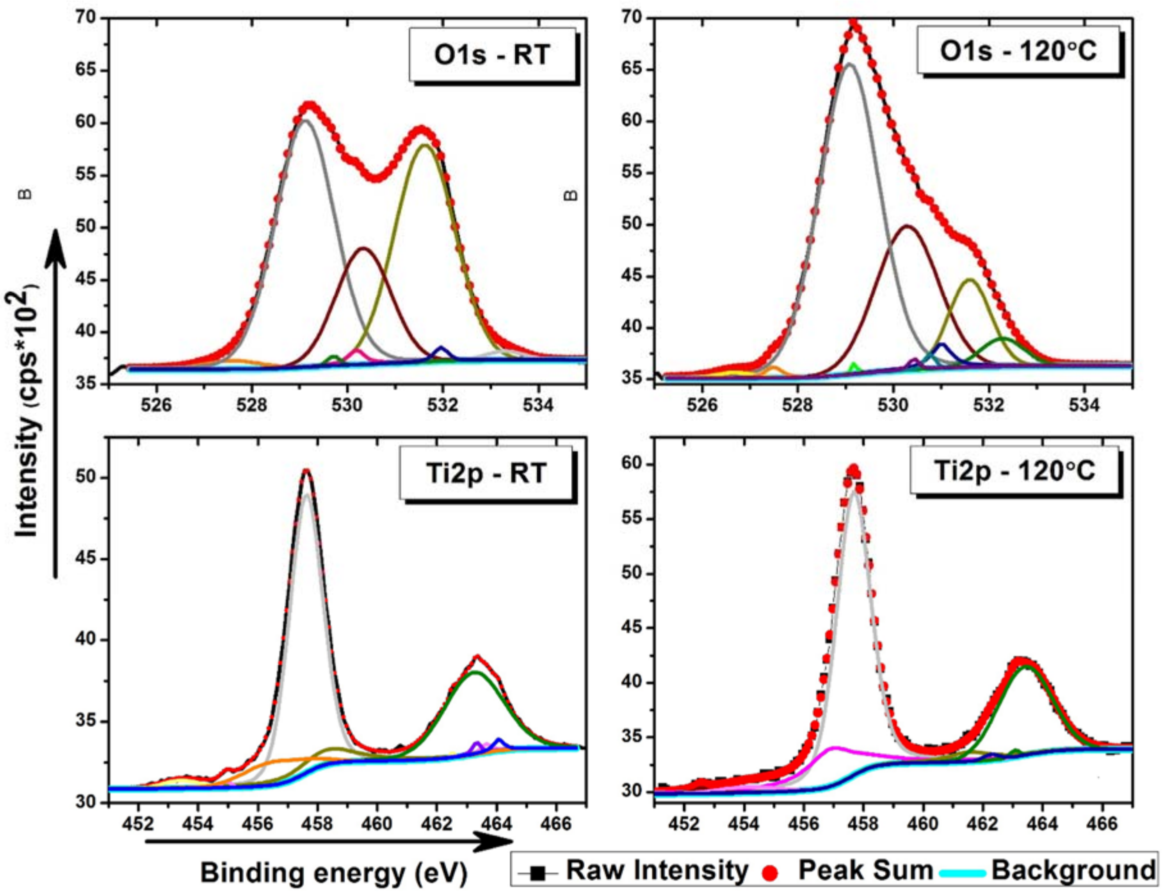

Figure S1: Comparative DE convoluted O1s and Ti2p core level spectra for PS-P-TiO2 MOF co-crystallized in self-assembled polymer matrix formed at 24 and 120°C (suspensions in ethanol)

Figure S2

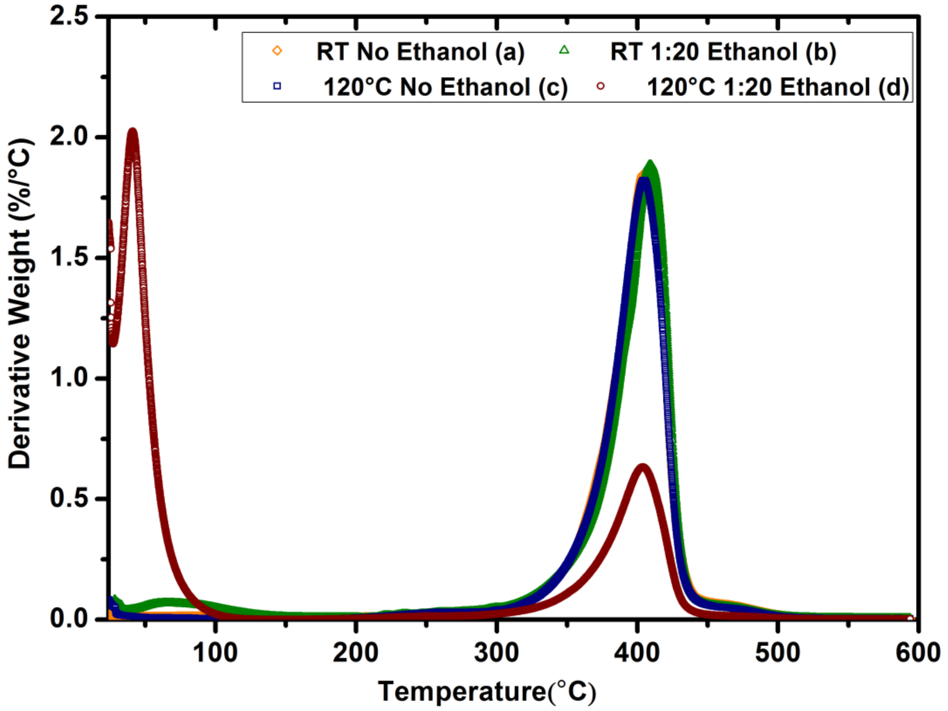

Figure S2: Differential scanning calorimetry (DSC) analysis of PS-P-TiO_2

Figure S3

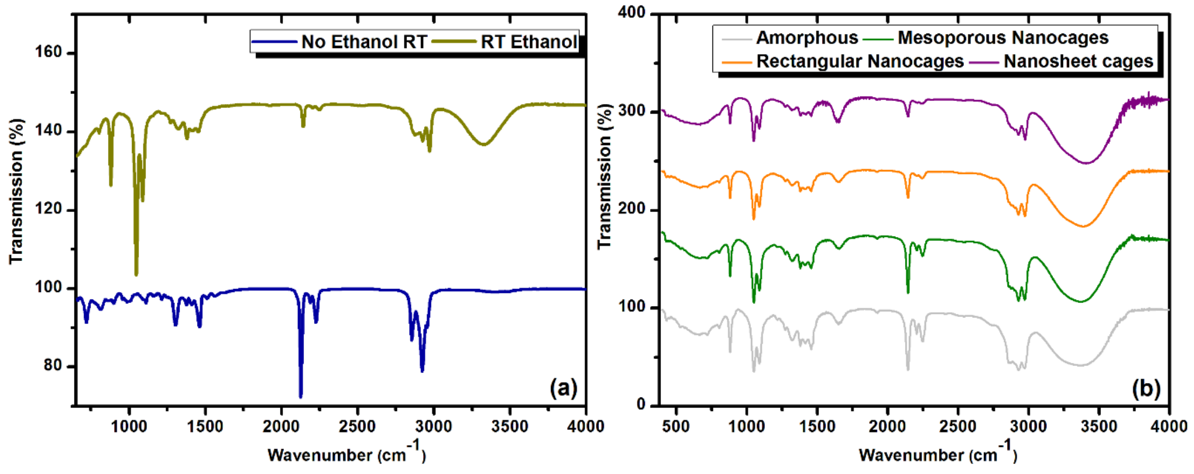

Figure S3: Comparison of the FT-IR spectra of PS-P-TiO2 complex in the (a) absence and presence of ethanol at RT and (b) presence of ethanol at 120°C with their respective morphologies.

Figure S4

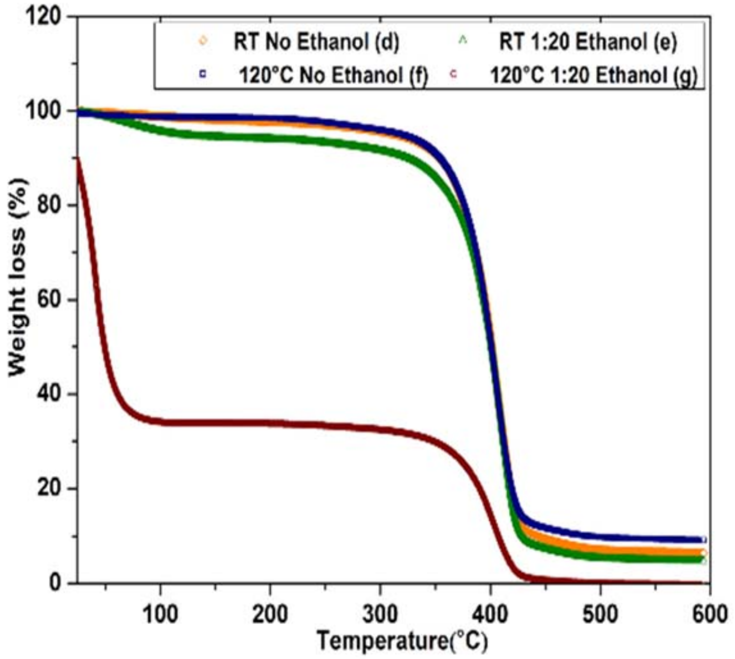

Figure S4: Fig (d–g) thermogravimetric analysis (TGA) at 120°C presents a two-stage decomposition profile indicating in (g) the release of bound water after crystallization at 120°C in the presence of ethanol

Figure S5

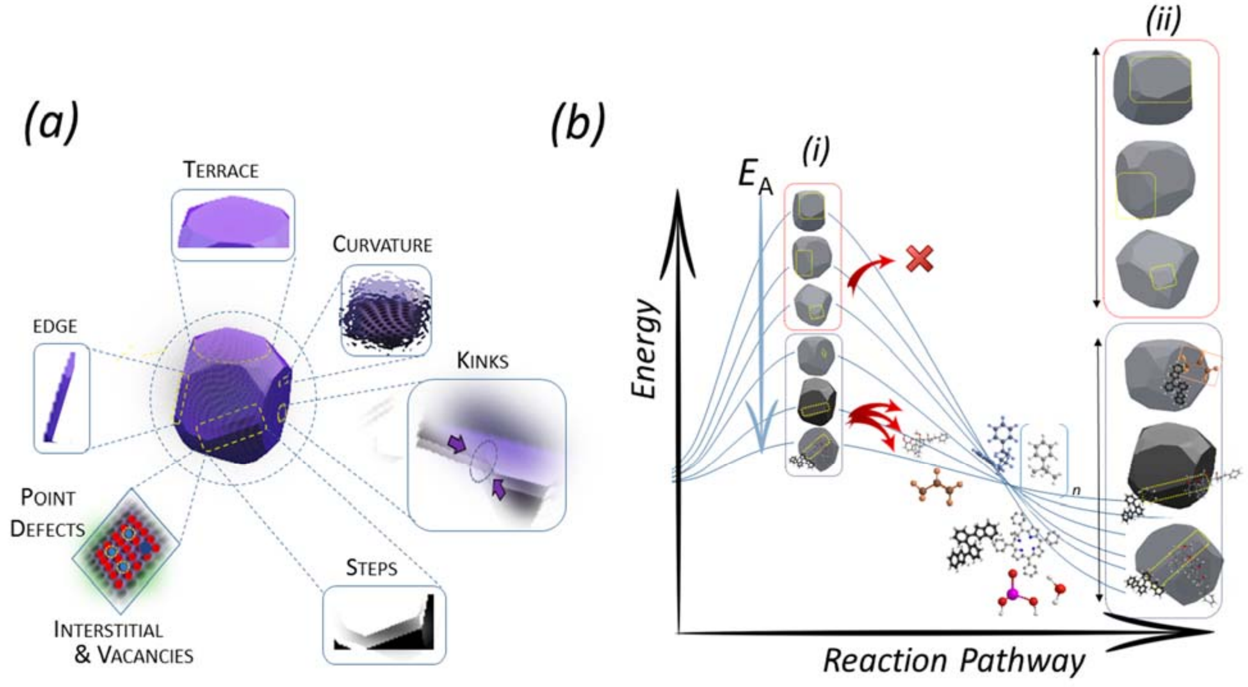

Figure S5. A Schematic illustration showing the energy driven surface construction of defect states at the catalytic metal oxide-IL interface.

(a) Defect sites on metal oxide surfaces are an important site for chemisorption of interacting molecules and a platform for 'evolutionary chemistry' to occur on the synthetic scale. (b) The energy-reaction pathway profile showing the role of edges, kinks and vacancy sites in the catalytic assembly and disassembly of shape and size dependent chemical structures. If energetically tuned, such surfaces can lower activation energies (E_A) to construct and coordinate structures around emerging heterogeneous nucleation sites causing growth arrest. (ii) is an enlarged version of (i).

Figure S6

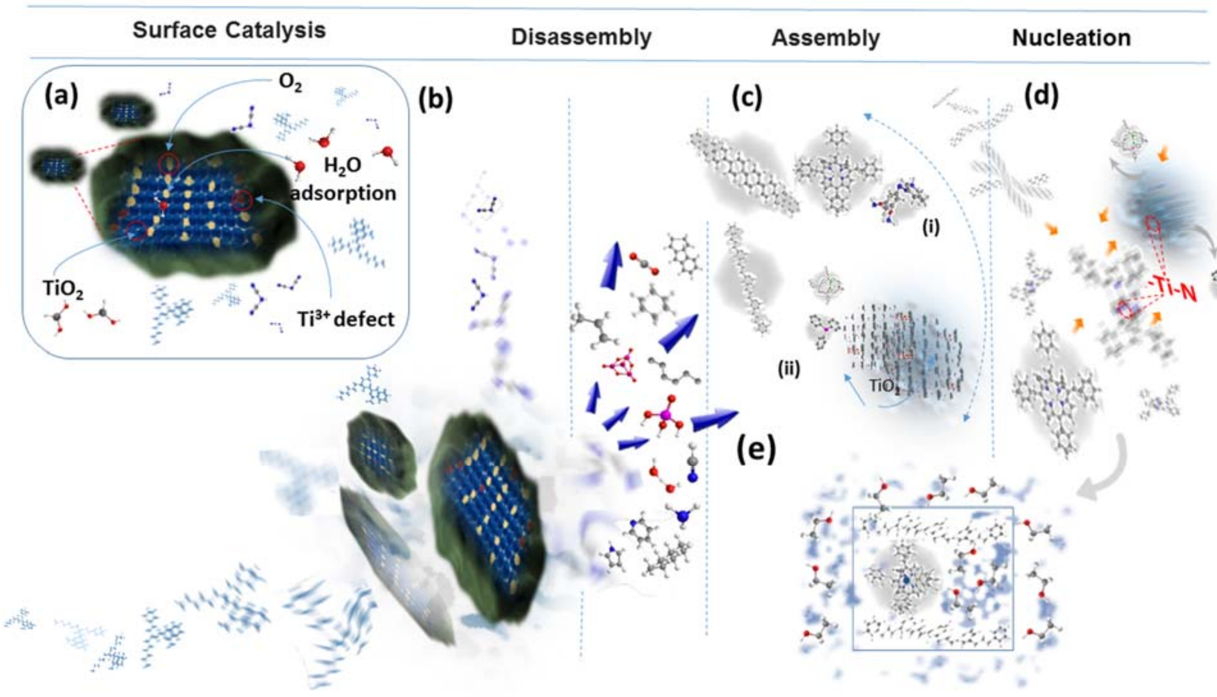

Figure S6 A schematic representation of the biomimetic assembly of PS-P-TiO_2

(a) (a) TiO_2 surface driven catalysis of interacting structures from the reaction medium is essentially defect directed and formation of (b) primary building blocks formed from the catalytic disassembly of chemical components from bulk reaction is determined by the interfacial surface energy of defect states that may or may not permit the (c) assembly of pre-cluster formation and (d) nucleation at the polymer-TiO_2 interface. Cluster orientated complexation by interfacial forces at nucleation sites lead to (e) polymer ‘caged’ TiO_2 at the quantum scale.

Figure S7

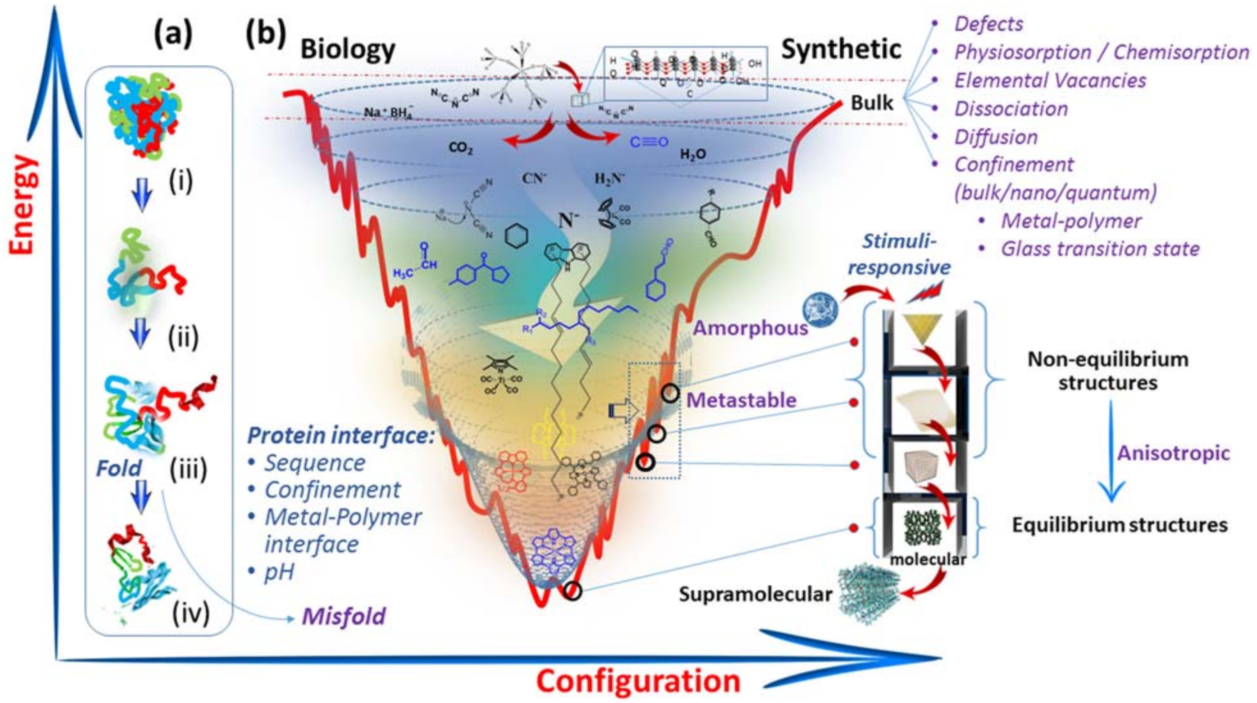

Figure S7 Energy landscape comparison between protein and synthetic assembly

A comparative vision of physical and chemical parameters controlling the energy landscape trajectory of protein and synthetically driven assemblies from unstable (high energy) to stable (low energy) states. The interactions of biological and synthetic surfaces are paralleled by kinetic pathway selectivity at catalytic surfaces and the dependency on charge, size and shape associations and the thermodynamic arrest of non-equilibrium states. Such metastable geometries intermediate between extreme energy states form the basis of tuneable materials with stimuli-responsive properties.

Figure S8

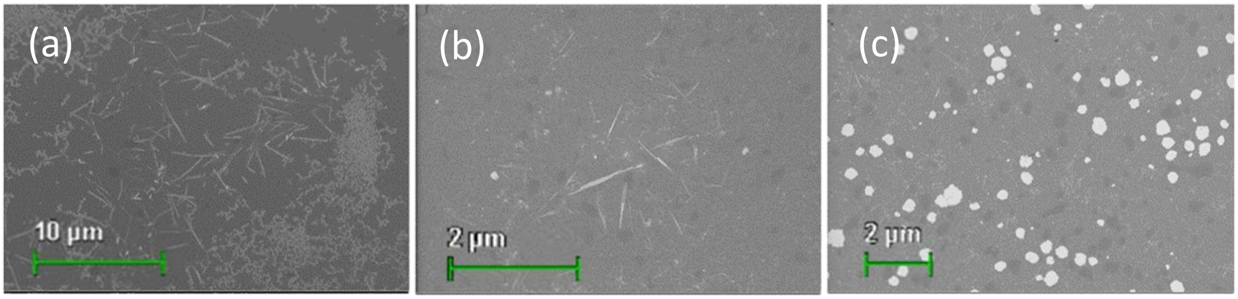

Figure S8 SEM images of (a) self-assembled amorphous phase PS-P-TiO_2. Solvent (ethanol) responsive PS-P-TiO_2 reveals the morphological change of polymer matrix shown by the shape and size change of the polymer by the (b) disappearance of polymer strands to the appearance of (c) dense spherical polymers in 20-fold diluted samples. Scale bar as shown.

Figure S9

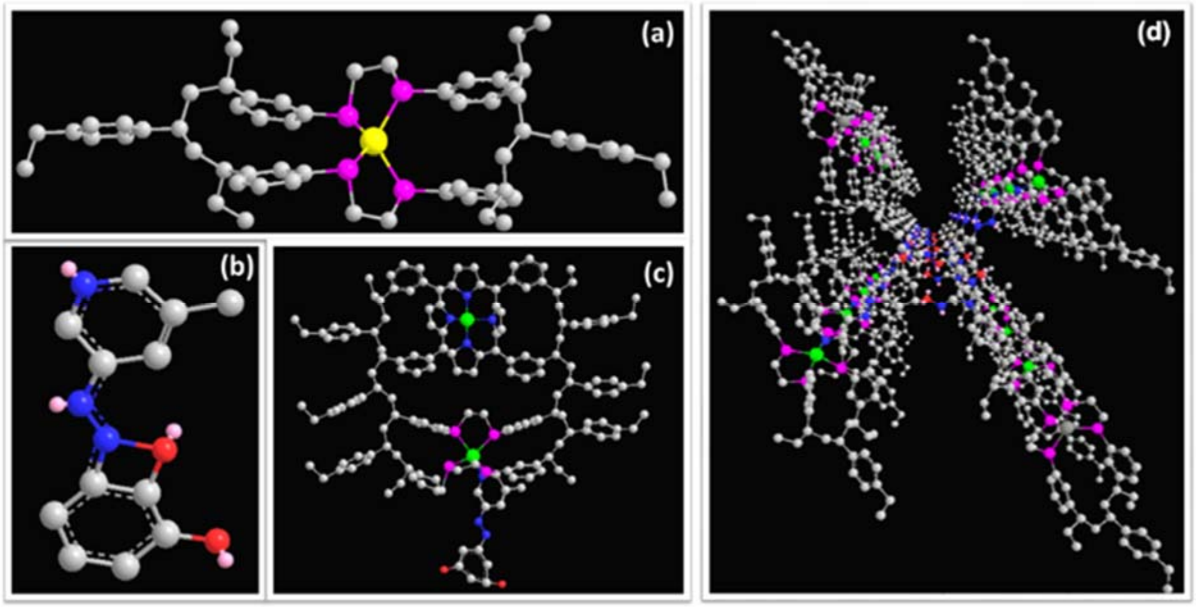

Figure S9 Geometrically stabilized individual structural units modelled by MM2 force field calculations showing (a) polystyrene with (1,2 ethanediyl)diphenyl phosphorane oxide ligand (b) (4-methyl-2-pyridylazo)phosphine oxide as the potential linker molecule and (c) a single polymer unit showing polystyrene conjugated with two ligands (i) titano porphyrin and (ii) (1,2 ethanediyl)diphenyl phosphorane oxide conjugated with Ti. The overall self-assembled metal organic framework is shown in (d).

Figure S10

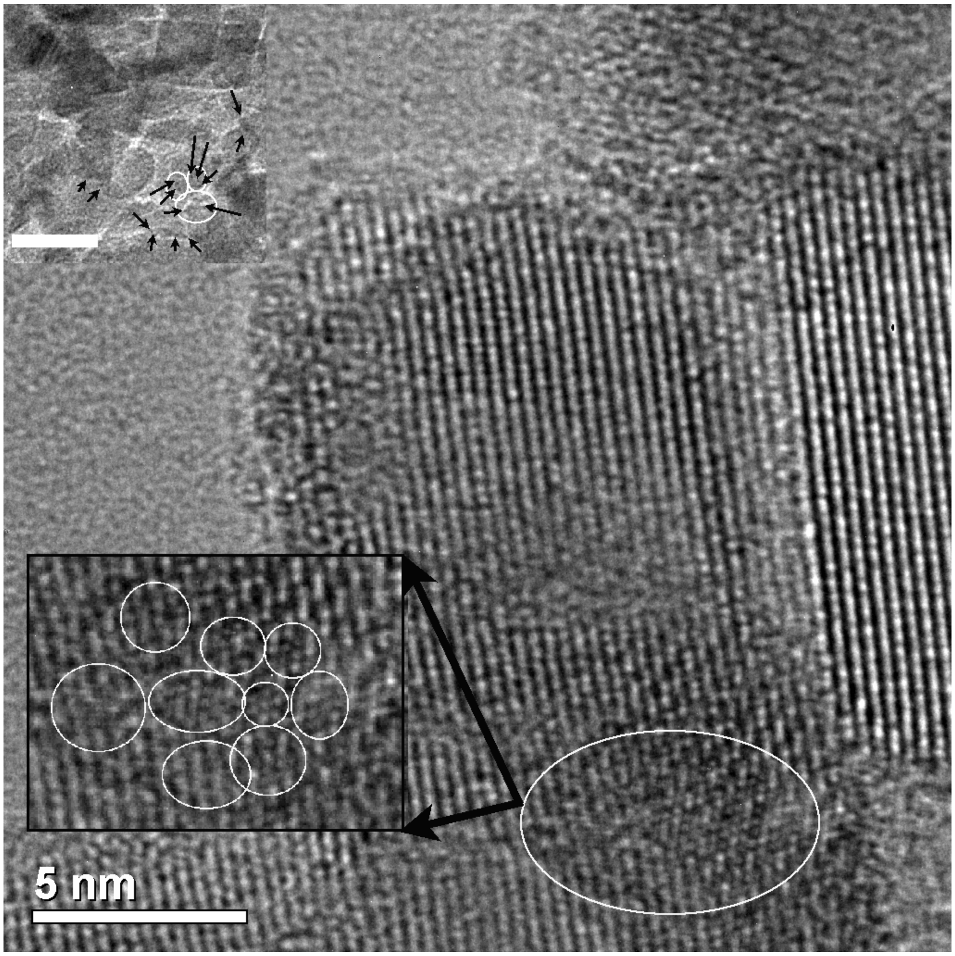

Figure S10: HRTEM atomic level bright field image showing the deposition on TiO_2 QDs exhibiting QDs regions are shown encircled areas.

Figure S11

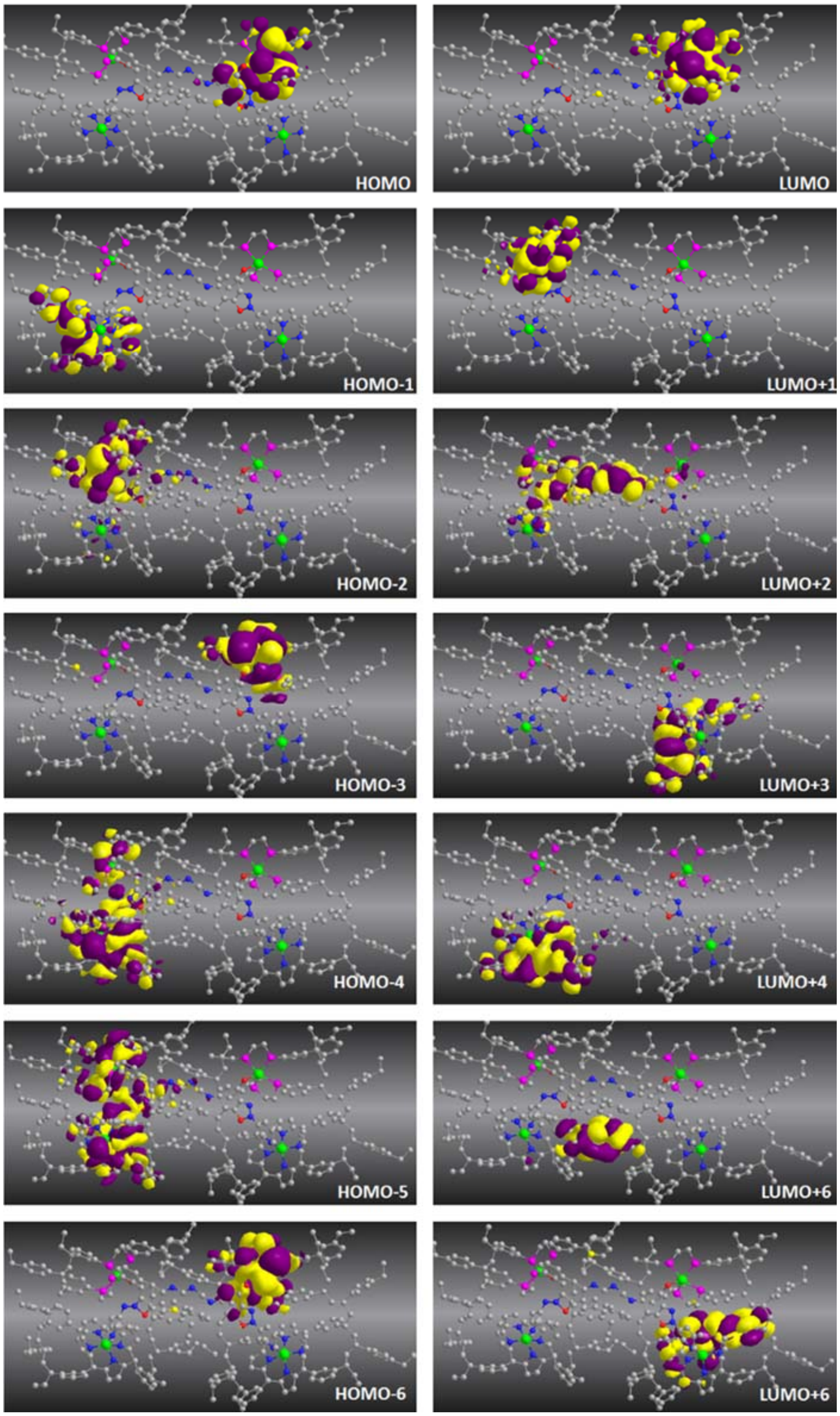

Figure S11: HUMO-LUMO orbital MM2 modelling showing the overlap between conjugated states of organic and metal oxide electron densities

Figure S12

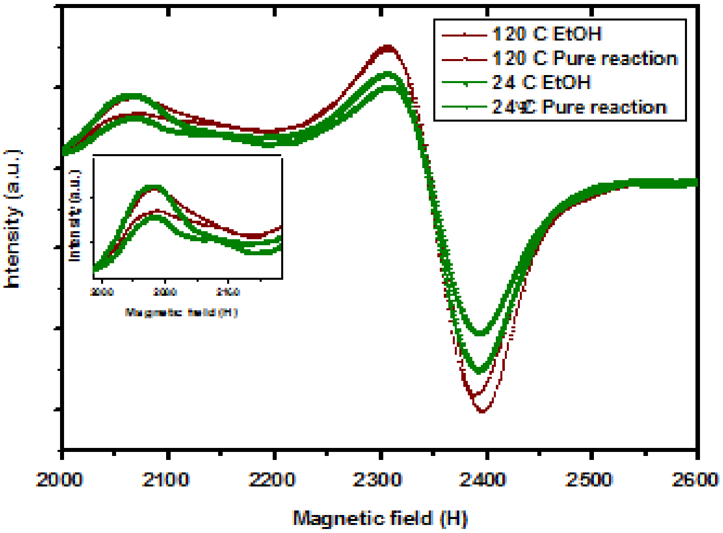

Figure S12 Spin state measurement of quantum confined PS-P-TiO_2

Electron paramagnetic resonance (EPR) of PS-P-TiO_2 QDs characteristic of an anisotropic nature of the spin state of free radicals

The detection of symmetric and asymmetric peaks of variable depth in the EPR spectra shown in Fig S9 is characteristic of the anisotropic nature of the spin state of free radicals and unpaired electrons of nuclei which is dramatically altered by magnetic interactions from other sources within the chemical lattice(s) or material surroundings. The association of resonating signals manifest as 'splitting' of the spectral signals which is less erratic at 120°C relative to the lower temperature and results in narrowing of the spectral width in the presence of ethanol. Establishing the surface coordination of solvent to TiO_2 formed from a colloidal suspension of ethanol / water (Koelle et al, 1985) and attempts to identify paramagnetic species in the hydrated state of TiO_2 is supported by the formation of $Ti^{4+}O\bullet–Ti^{4+}OH^{-}$ evidenced by a low-temperature irradiated state EPR study (Howe and Gratzel, 1987). The transient nature of the Ti^{4+} coupled hydroxyl moiety (Howe et al, 1987) liberating $Ti^{4+}O\bullet^{+}$ leads to its availability to generate methanol radicals $\bullet CH_2OH$ (Hurum et al, 2003) on the surface of TiO_2 that correspond to the phase sensitive g factor spectral reading of 2.004 at 18 G. This value corresponds to the same order of the asymmetrical peak seen in the presence of ethanol (g = 2.009 at 10 G) in the present study at 120°C but differs from the RT g factor value of 2.0133 also in ethanol suggesting that $\bullet CH_3CH_2OH$ radical formation may be impeded by the surface characteristics of TiO_2 in the absence of thermal annealing. The change in the spin state of the symmetrical peak corresponding to the g factor derivative of 1.976 and 1.977 of the nanocaged structures in the rutile phase in the absence and presence of ethanol respectively after thermal annealing is equivalent to the same effect observed for rutile under visible illumination assigned to a g factor of $1.975^{\perp}$ (Hurum et al, 2003). The equivalency reported by Hurum et al extends to the size uniformity of nucleating rutile morphologies that elicit quantum scale-like spin-lattice characteristics anticipated for nanocaged rutile TiO_2 resulting in superior (1) charge transfer properties (2) photo active enhancement through increased photon absorption to the visible range and (3) lowering of the conduction-valence band gap compared to the anatase phase as reported in their investigation. However, EPR measurements for samples above a 5-fold dilution in ethanol did not generate any spectral signals probably due to the diminishing effect of the solvent driven colloidal phase.

References

Koelle, U.; Moser, J.; Graetzel, M., *Inorganic Chemistry* **1985,** *24* (14), 2253-2258. DOI 10.1021/ic00208a026.
78. Howe, R. F.; Gratzel, M., *The Journal of Physical Chemistry* **1987,** *91* (14), 3906-3909. DOI 10.1021/j100298a035.
79. Hurum, D. C.; Agrios, A. G.; Gray, K. A.; Rajh, T.; Thurnauer, M. C., *The Journal of Physical Chemistry B* **2003,** *107* (19), 4545-4549. DOI 10.1021/jp0273934.

Tables

Table S1

24°C 1-10		120°C			
		1-10		1-20	
Polystyrene	TiO2	Polystyrene	TiO2	Polystyrene	TiO2
**Lattice parameter**<br>Isotactic, Hexagonal a=b=21.2288 Å c=6.65Å	**Lattice parameter**<br>Rutile Tetragonal a=4.6047Å, b=2.9092Å	**Lattice parameter**<br>Syndiotatic, Orthorhombic a=8.3279 Å, b= 29.7045 Å, c=5.0888 Å	**Lattice parameter**<br>Rutile Tetragonal a= 4.6087 Å, c= 2.7443 Å	**Lattice parameter**<br>Syndiotatic, Orthorhombic a=7.9511 Å, b= 27.6587 Å, c=5.05964 Å	**Lattice parameter**<br>Rutile Tetragonal a=4.5560Å c=3.0368Å
H(220)=15.73% H(410)=20.08%	R(100)-30.2%	O(001)=12.35% O(121)=17.20%	R(100)-16.89%	O(121)=29.29% O(141)=26.80%	R(100)-23.75%
H_{total}= 51.13%	R_{total}= 48.87%	O_{total}= 66.01%	R_{total}= 33.99%	O_{total}= 76.25%	R_{total}= 23.75%
**Particle size**<br>Majority 2-3nm Minority (2%) 9nm	**Particle size**<br>Majority 1.25-2.81 nm Minority sub-nanometer (7%)	**Particle size**<br>Majority Sub nm(21%)-5.6nm Minority (7%) 7-14nm	**Particle size**<br>Majority Subnanometer to 4nm	**Particle size**<br>Majority 1.7-4.1nm Minority (2%) 7-11nm (2.6%)	**Particle size**<br>Majority 2.81 nm Minority none

Table S1: Lattice parameters and particle size of polystyrene and TiO2 in PS-P-TiO_2 (TiO_2 MOF co-crystallized in the polystyrene matrix) at 24°C (10-fold ethanol) and 120°C (10 and 20-fold ethanol).

Table S2

P2p							
120˚C				24˚C			
Binding Energy (eV)	Area (%)	FWHM (eV)	Structure	Binding Energy (eV)	Area (%)	FWHM (eV)	Structure
129.51	3.3	0.7	Ti-P	130.16	2.7	0.958	P
130.06	2.9	0.6	P	130.81	0.7	0.491	$[P(C_6H_5)_3]$
130.82	5.5	0.8	$[P(C_6H_5)_3]$	131.21	1.7	1.28	$[P(C_6H_5)_3]$
131.81	6.4	0.9	$[P(C_6H_5)_3]$	132.12	66.4	0.452	$[P(C_6H_5)_3]$
132.30	75.4	1.6	$[(CH_3CH_2)(P(O(C_6H_5))_2]$	132.50	0.5	0.178	$PO(C_6H_5)_3$
133.35	2.5	0.7	$Ti_3(PO_4)_4$	133.01	27.2	1.191	Na_3PO_4
134.32	2.1	1.0	H_3PO_3	133.78	0.4	0.488	$PO_4(C_6H_5)_3$
135.60	1.9	1.0	P_2O_5				
N1s							
120˚C				24˚C			
394.85	1.0	0.6	CH_3-(C_5H_3N)-N=N-(C_6H_3)(OH)(OH)	395.77	2.5	1.1	
396.19	5.3	1.4	Ti-N-C	396.70	8.0	1.1	Ti-N-C
397.47	48.9	1.4	TiN0.12O0.98	397.59	45.1	1.2	TiN0.09O0.74
397.77	0.2	0.2	TiN	398.18	1.2	0.4	$[OP(C_6H_5)(NH_2)_2]$
398.58	37.5	1.6	$C_{44}H_{30}N_4$	398.73	25.4	1.2	$C_{44}H_{30}N_4$
399.00	1.3	0.4	$C_6H_5CHNC_6H_5$	399.19	17.2	2.1	$(C_6H_5NC_6H_4)n$
399.38	1.2	0.4	$(C_6H_5NC_6H_4)n$	400.39	0.2	0.2	$C_6H_4(CH_3)NH_2$
400.07	4.6	1.8	$(C_6H_5N)_2$	400.97	0.4	0.4	$(-C_6H_5NH-)n$
C1s							
120˚C				24˚C			
282.85	1.6	1.3	C_2H_4	283.12	2.8	1.3	CO
283.33	1.0	0.9	CO	284.50	75.4	1.2	$(C_8H_8)_n$
284.28	0.6	0.4	$CH_2=C(CH_3)COOCH_3$	285.54	20.1	1.8	$C_{44}H_{30}N_4$
284.50	76.0	1.3	$(C_8H_8)_n$	287.51	1.7	2.8	$(-C_6H_5NH-)n$
285.52	17.7	1.7	$C_{44}H_{30}N_4$				

Ti2p							
120˚C				24˚C			
Binding Energy (eV)	Area (%)	FWHM (eV)	Structure	Binding Energy (eV)	Area (%)	FWHM (eV)	Structure
452.55	0.60	0.55	$TiSi_2$	453.50	2.0	1.7	Ti
454.12	2.17	2.23	TiN0.12/TiN0.23(2p3/2)	454.93	0.4	0.4	TiN(2p3/2)
456.89	13.90	1.95	Ti_2O_3(2p3/2), Ti -tetraphenylporphyrin (TTP)	456.25	6.0	2.0	TiO (2p3/2)
457.65	57.64	1.36	Ti_2O_3(2p3/2), Ti -tetraphenylporphyrin (TTP)	457.60	51.0	1.3	Ti_2O_3(2p3/2)
461.56	2.85	1.75	TiO (2p1/2)	458.06	9.6	2.4	TiO_2(2p3/2)
462.26	0.60	0.64	Ti_2O_3(2p3/2)	461.96	5.5	1.2	Ti_2O_3(2p3/2)
463.06	0.31	0.37	$[Ti(NO)_2(P(C_6H_5)_3)_2]$	462.57	7.6	0.8	TiN
463.39	23.90	2.11	Ti2O3 (2p1/2)	463.13	2.3	0.6	$[Ti(NO)_2(P(C_6H_5)_3)_2]$
				463.62	11.2	1.2	Ti_2O_3(2p3/2)
				464.37	4.4	1.5	Ti_2O_3(2p3/2)
O1s							
120˚C				24˚C			
526.68	0.6	0.9	CH3-(C5H3N)-N=N-(C6H3)(O)(O))(CH3-(C5H3N)-N=N-(C6H3)(O)(OH)	527.61	1.3	1.4	CH3-(C5H3N)-N=N-(C6H3)(OH)(OH)
527.49	0.8	0.4	CH3-(C5H3N)-N=N-(C6H3)(OH)(OH)	529.11	42.2	1.5	TiO_2
529.08	55.9	1.5	TiO_2	529.71	0.3	0.3	TiO_2
529.19	0.2	0.1	Ti_2O_3	530.19	1.0	0.4	TiO_2
530.29	25.5	1.5	TiO_2	530.34	17.6	1.4	TiO_2
530.43	0.4	0.2	TiO_2	531.62	36.0	1.5	$TiO_{0.9}$
530.77	0.5	0.3	O2/Ti	531.95	0.7	0.4	$TiO_{0.73}$
531.01	2.1	0.5	TiO_2	533.30	1.0	1.0	$O/(-C_6H_5N-H)n$
531.60	10.6	1.0	$TiO_{0.9}$				
532.28	3.4	1.1	P_2O_5				

Table S2: Comparative DE convoluted O1s and Ti2p Core level spectra for PS-P-TiO_2 (TiO_2 MOF co-crystallized in the polystyrene matrix) formed at 24 and 120°C (suspensions in ethanol).

Table S3

Samples	Peak	Position BE (eV)	FWHM (eV)	Raw Area (cps eV)	Atomic Mass	Atomic Conc %	Mass Conc %
RT QD	P 2p	130	1.674	1047.6	30.974	1.89	4.31
	C 1s	282.2	1.384	27902.2	12.011	78.24	69.35
	N 1s	395.4	2.346	4213.3	14.007	7.28	7.53
	Ti 2p	455.3	1.347	4007.1	47.878	1.68	5.93
	O 1s	527	3.86	9095.6	15.999	10.91	12.88
120 QD	P 2p	129.9	1.706	1144.2	30.974	2.17	4.83
	C 1s	282.3	1.387	26290.3	12.011	77.75	67
	N 1s	395.4	2.307	3642	14.007	6.64	6.67
	Ti 2p	460.9	2.089	6012.3	47.878	2.65	9.12
	O 1s	526.9	2.111	8530.6	15.999	10.79	12.39

Table S3: Elemental analysis by XPS indicating presence of TiO_2 at RT and Ti_2O_3 at 120°C

Table S4

Transition Glass Temperature (T_g)				Fragility Index (T_m/T_g)					
$[(polystyrene)_m]/[(PS–P–TiO_2)_{Tg}]$				$[(PS–P–TiO_2)_{Tg}/(polystyrene)_{Tg}]$				$[(T_m/T_g)_{bulk\ PS}] / [(T_m/T_g)_{(PS–P–TiO2)}]$	
120°C		RT		120°C		RT		120°C	RT
Ethanol (20-fold)	Neat	Ethanol (20-fold)	Neat	Ethanol (20-fold)	Neat	Ethanol (20-fold)	Neat	Ethanol (20-fold)	Ethanol (20-fold)
0.72 (240/333.6)	0.57 (240/420)	0.57 (240/420)	0.56 (240/423.6)	3.34 (333.6/100)	4.20 (420/100)	4.24 (423.6/100)	4.24 (423.6/100)	3.30 (2.4/0.72)	4.21 (2.4/0.57)

Table S3: Comparison of the fragility index and transition glass temperature of polystyrene in PS-P-TiO_2 (TiO_2 MOF co-crystallized in the polystyrene matrix) from bulk polystyrene in the presence and absence of ethanol at RT and 120°C respectively. The transition glass temperature of PS-P-TiO_2 was determined by DSC experiments.

Table S5

UV Peak Position	Molecular Orbital Transition (MM2 force field)		Photo Luminescence		Molecular Orbital Transition (MM2 force field)	
(eV/nm)	Energy (eV)	HOMO-LUMO	(eV/ nm)	Area%	Energy (eV)	HOMO-LUMO
**4.33 / 286**	**-6.933 – (-2.546) = -4.387**	**HOMO-6 – LUMO +6**	**3.5895 / 345.41**	**0.00**	**-**	**-**
**2.78 / 446**	**-5.380 –(-2.546) = -2.834**	**HOMO-1 – LUMO +6**	**3.5096 / 345.41**	**0.02**	**- 6.435-(-2.918)= -3.517**	**HOMO-4 – LUMO +4**
**2.07 / 600**	**-5.042 – (-2.918) = -2.124**	**HOMO – LUMO +4**	**3.5065 / 353.59**	**0.10**	**-**	**-**
**1.68 / 737**	**-5.380 – (-3.688) = -1.692**	**HOMO-1 – LUMO +2**	**3.4234 / 362.17**	**2.30**	**-6.315-(2.918) = -3.40**	**HOMO-3 – LUMO +4**
**1.36 / 912.5**	**-5.042 – (-3.688) = -1.354**	**HOMO – LUMO +2**	**3.3202 / 373.42**	**5.70**	**-6.9333 -(-3.667) =-3.27**	**HOMO-6 – LUMO +3**
**1.16 / 1070**	**-5.042 – (-3.856) = -1.186**	**HOMO – LUMO +1**	**3.1285 / 396.31**	**39.00**	**-5.999- (- 2.546) =-3.053**	**HOMO-2 – LUMO +6**
**-**	**-**	**-**	**2.8699 / 432.02**	**22.76**	**-5.380 - (2.545) =2.83**	**HOMO-1 – LUMO +6**
**-**	**-**	**-**	**2.6544 / 467.08**	**24.25**	**-5.087-(-2.485) =-2.638**	**HOMO –1– LUMO +5**
**-**	**-**	**-**	**2.3646 / 524.33**	**3.44**	**-5.042-(-2.742) =2.3**	**HOMO – LUMO +5**

Table S5: Correlation of UV and photoluminescence (PL) peak positions with molecular orbital transitions obtained from the structures stabilized by MM2 force field and MO calculations based on Extended Huckel model.
